# Plasma-Derived Extracellular Vesicles as Systemic Mediators of Radiation Response and Radiation Mitigation by Activated Protein C in a Rat Model

**DOI:** 10.3390/antiox15091160

**Published:** 2026-09-11

**Authors:** Shivani Bansal, Sunain Deol, Meth Jayatilake, Yaoxiang Li, Brian L. Fish, Xiao Xu, Jose A. Fernandez, John H. Griffin, Tracy Gasperetti, Meetha Medhora, Marjan Boerma, Heather A. Himburg, Amrita K. Cheema

**Affiliations:** 1 Department of Oncology, Georgetown University Medical Center, Washington, DC 20007, USA; sm3451@georgetown.edu (S.B.); skd74@georgetown.edu (S.D.); mmj61@georgetown.edu (M.J.); yl814@georgetown.edu (Y.L.); 2 Department of Radiation Oncology, Medical College of Wisconsin, Milwaukee, WI 53226, USA; bfish@mcw.edu (B.L.F.); tgasperetti@mcw.edu (T.G.); medhoram@mcw.edu (M.M.); hhimburg@mcw.edu (H.A.H.); 3 Department of Translational Medicine, Scripps Research Institute, La Jolla, CA 92037, USA; xxu@scripps.edu (X.X.); jfernand@scripps.edu (J.A.F.); jgriffin@scripps.edu (J.H.G.); 4 Division of Radiation Health, Department of Pharmaceutical Sciences, University of Arkansas for Medical Sciences, Little Rock, AR 72205, USA; mboerma@uams.edu; 5 Department of Biochemistry and Molecular & Cellular Biology, Georgetown University Medical Center, Washington, DC 20007, USA; 6 Department of Radiation Medicine, Georgetown University Medical Center, Washington, DC 20007, USA

**Keywords:** extracellular vesicles, radiation injury, metabolomics, lipidomics, biomarkers, activated protein C (APC), WAG/RijCmcr rat

## Abstract

Radiological emergencies necessitate biomarkers that not only estimate absorbed ionizing radiation (IR) dose but also guide timely interventions to prevent or delay multi-organ injury. Conventional LC–MS-based metabolomics of bulk plasma is constrained by matrix effects that mask low-abundance species. Extracellular vesicles (EVs) constitute a metabolically enriched, underexplored compartment that can provide complementary insight into systemic metabolic and redox responses to IR. Female WAG/RijCmcr rats were exposed to 13.0 Gy leg-out partial-body X-rays and treated with one of three activated protein C (APC) variants—rat wild-type (WT), rat 3K3A-APC, or human WT APC—administered 24- and 48 h post-irradiation. Longitudinal plasma collections (days 1, 14, 30, and 90) were subjected to metabolomic and lipidomic profiling of whole plasma and matched EV-enriched fractions to define signatures of acute radiation syndrome (ARS) and delayed effects of acute radiation exposure (DEARE), and their modulation by APC. ARS was marked by early dyslipidemia and widespread metabolic disruption, evolving into DEARE with persistent alterations in energy metabolism, and nucleotide biosynthesis, consistent with sustained oxidative and inflammatory stress. EV profiles showed matrix-specific, time-dependent trajectories distinct from plasma, with prominent lipid dysregulation and enrichment of fatty acid β-oxidation, sphingolipid, and cholesterol pathway metabolites at day 90. Rat 3K3A-APC promoted early EV metabolic normalization, whereas rat WT APC more effectively mitigated late DEARE-associated changes. Elevated sphingomyelins in plasma EVs at day 90 may suggest a compensatory or anti-inflammatory lipid response. These findings suggest that plasma-derived EVs may provide a sensitive matrix for radiation biomarker discovery and may help elucidate APC-mediated modulation of IR-induced metabolic and redox disturbances.

## 1. Introduction

Partial or whole-body exposure to ionizing radiation (IR) at acute, large doses (>2 Gy), can potentially be lethal if not diagnosed and treated swiftly. In individuals who survive the acute radiation syndrome (ARS), delayed effects of radiation exposure (DEARE) can lead to life-threatening multiple-organ injuries, at least in part due to progressive damage to the vascular endothelium [1]. Early characterization of metabolic perturbations that precede and accompany DEARE is therefore critical for the development of effective medical countermeasures and biodosimetry tools. In this context, the discovery of metabolomics-based biomarkers represents a promising strategy. This approach uses high-throughput qualitative and quantitative profiling of metabolic signatures to predict late effects of IR [2,3]. Historically, urine and plasma have been the most extensively characterized matrices for post-IR metabolic profiling; however, other matrices, such as tissues, feces, and saliva, have also been explored [4,5]. The discovery of novel IR injury biomarkers could be beneficial for the early prediction of adverse effects, as well as for monitoring response to radioprotectors and/or mitigators [6,7,8]. Previously, we have reported IR-induced alterations in urinary metabolic profiles using the WAG/RijCmcr adult female rat model, identifying biomarkers associated with oxidative stress, dyslipidemia, disrupted energy metabolism, and DNA damage [9]. However, in conventional biofluid profiling, low-abundance metabolites are frequently masked by highly abundant species and matrix effects, including ion suppression, which can limit biomarker discovery. Extracellular vesicles (EVs) are nano-sized particles released by most cell types under physiological and pathological conditions and are readily isolated from common biofluids. EVs are crucial mediators of intercellular communication, carrying a diverse cargo of lipids, proteins, nucleic acids, and metabolites [10,11,12]. The detection potential of EV metabolites is also enhanced by the increased stability of EVs against nucleases and proteases due to the presence of the lipid bilayer [13]. Consequently, metabolomics and lipidomics-based characterization of EV content could yield us biomarkers that may otherwise be unstable or obscured in plasma or other matrices. Hence, EVs are gaining credence as an important matrix for the discovery of novel biomarkers of IR injury and for validating clinically relevant biomarkers in health and disease progression, including cancer [14]. Furthermore, several recent studies have identified cancer-associated EV signatures [15,16]. However, most comparative EV studies to date have emphasized proteomic and transcriptomic changes, whereas metabolomics-driven biomarker discovery in EVs, particularly in the setting of IR exposure, remains relatively underexplored [17]. We have previously optimized an experimental workflow that enriches exosomal fractions from peripheral blood plasma, enabling detection of low-abundance biomolecules that are typically below the limit of detection in unfractionated plasma [18]. We have demonstrated IR-induced pathophysiological perturbations with special emphasis on the role of plasma-derived EVs as an important matrix for the identification of biomarkers for IR injury. Moreover, in a recent study, we showed that EVs isolated from small urine volumes (0.5 mL) can yield informative biomarkers of IR exposure [19].

Activated protein C (APC) is a vitamin K-dependent serine protease with anticoagulant and anti-inflammatory properties, which has been shown to improve endothelial function, enhance survival after irradiation, and attenuate late organ injury in preclinical models [20,21]. Our previous work demonstrated that recombinant APC variants exert significant mitigation of IR effects, alleviating ARS and DEARE [21]. Because emerging data indicate that many benefits of APC are independent of its anticoagulant activity, the recombinant 3K3A-APC mutant was engineered to retain full cytoprotective function while exhibiting markedly reduced anticoagulant activity (<10% of WT APC) [22,23]. Consistent with this, mice with genetically elevated APC (APCHi) display reduced radiation injury, and several lipids and metabolites dysregulated in WT animals are near sham levels in the APCHi mice [24].

Herein, we enriched the exosomal fraction of plasma samples collected from WAG/RijCmcr female rats longitudinally, starting from day 1 through 3 months after exposure to 13.0 Gy of leg-out partial body irradiation (PBI). Separate cohorts of rats received rat WT APC, rat 3K3A APC, or human WT APC as two bolus injections at 24 and 48 h post-irradiation. This model permits serial sampling and analysis of plasma-derived EVs, thereby enabling assessment of structural and functional alterations over time and evaluation of potential mitigation of IR effects by APC.

The aims of this study were twofold: (i) to identify significantly dysregulated biomarkers of IR exposure in plasma-derived EVs and assess their overlap with the plasma metabolome, and (ii) to delineate the extent to which APC variants mitigate IR-induced metabolic and lipidomic perturbations. To our knowledge, this is the first LC-MS/MS-based metabolomic and lipidomic investigation of IR-induced biomarkers in the WAG/RijCmcr rat model, with direct comparison of plasma and matched plasma-derived EVs. The study reveals EV-specific features and long-term metabolic trends that are frequently obscured in bulk plasma, supporting EV-focused metabolomics as a sensitive platform for biomarker discovery and for evaluating interventions such as APC.

## 2. Materials and Methods

### 2.1. Animal Care and Irradiation Protocols

#### 2.1.1. Animals and Groups

All animal protocols were approved by the Institutional Animal Care and Use Committees (IACUC) at the Medical College of Wisconsin, Milwaukee. WAG/RijCmcr female rats were irradiated at about 11–12 weeks of age (~155 gm). Rats were maintained in a dedicated barrier facility and provided with daily enrichment. All study rats were observed twice per day for clinical signs of distress. Rats were observed by institutional veterinary staff who were blinded to the treatment groups and by lab staff who were not blinded to the treatment groups. The rat cohorts were randomized into the following groups: (1) no irradiation, vehicle (*n* = 9); (2) 13 Gy leg-out PBI (*n* = 9); (3) 13 Gy leg-out PBI with human WT APC (*n* = 10); (4) 13 Gy leg-out PBI with rat WT APC (*n* = 10); and (5) 13 Gy leg-out PBI with variant rat 3K3A APC (*n* = 10). The dose of 13.0 Gy was selected based on the extensively characterized WAG/RijCmcr rat leg-out PBI model, in which one hind limb is externally shielded during irradiation to spare a small fraction (~5–8%) of the bone marrow [25,26]. This partial marrow sparing allows animals to survive the acute hematopoietic and gastrointestinal radiation syndromes (H-ARS and GI-ARS) that would otherwise be lethal after uniform total-body irradiation at this dose, while still producing sufficient systemic injury to generate the delayed effects of acute radiation exposure (DEARE) and prolonged survival necessary for follow-up, including pulmonary and renal injury, over the following weeks to months [25,26]. This dose has been used in our prior longitudinal urinary metabolomic study in this same model [9], allowing the present findings to be interpreted within an established injury trajectory. Consistent with this rationale, 13 Gy leg-out PBI produced measurable morbidity and mortality in the irradiated vehicle group (~10–30% mortality by day 120) without precluding longitudinal sample collection through day 90, thereby permitting assessment of both the acute (days 1–14) and early delayed (days 30–90) phases of radiation injury, and providing an adequately injured baseline against which the mitigating effects of APC variants could be evaluated [25]. Briefly, rats were restrained and irradiated without the use of anesthetics. One of the hind limbs of each rat was externalized carefully and shielded with a 0.25-inch lead block. An X-RAD 320 kVp orthovoltage X-ray system (Precision X-Ray, North Branford, CT, USA) was operated at 320 kVp and 13 mA with a half-value layer of 1.4 mm Cu and a dose rate of 1.69 Gy/min, for a total dose of 13 Gy. Radiation was delivered posterior-to-anterior to the rat. Dosimetry was performed as described by Medhora et al. [26]. All rats received supportive care, including hydration by daily subcutaneous injection of saline 40 mL/kg/day from days 2–10, antibiotics, and enrofloxacin (10 mg/kg/day) from days 2–14, given in the drinking water. Powdered food was added to the cages from days 35 to 70 after irradiation due to tooth loss that resolved by day 70.

#### 2.1.2. APC Treatment

Recombinant rat WT APC and the variant rat 3K3A-APC were prepared by Dr. Griffin’s laboratory (Scripps Research Institute, La Jolla, CA, USA) and these purified proteins plus purified plasma-derived human WT APC were used for this study. All three APC stock solutions were supplied as aliquots in a stabilization buffer (10 mM sodium citrate, 300 mM sodium chloride solution at a pH of 6.0). These APC drugs were stored at −80 °C until use. The APCs were administered to rats by intravenous (IV) injection at 24 and 48 h after irradiation. On the day of administration, the drugs were diluted to the appropriate concentration with Dulbecco’s phosphate-buffered saline as follows: rat WT APC (0.2 mg/kg), rat variant 3K3A APC (0.2 mg/kg), and human WT APC (0.6 mg/kg). The vehicle group received equivalent dilutions of stabilization buffer made in DPBS. Species-dependent differences in bioactivity potency are common and are well known for human versus murine APCs or 3K3A-APCs. As reported in the literature, human 3K3A-APC is approximately 2.5- to 5-fold more potent than murine 3K3A-APC with respect to its neuroprotective effects [27]. No extensive bioactivity comparisons are available for rat APCs versus human or mouse APCs. So, our dosing schedule is based on the assumption that the rat APCs would resemble mouse APCs for species-dependent differences compared to human APCs; hence, human APC was dosed at 0.6 mg/kg to be 3-fold higher than rat APC at 0.2 mg/kg for comparative bioactivity studies.

#### 2.1.3. Plasma Collection

Plasma samples were collected at 24 h, and at 14-, 30-, or 90-days post-irradiation, and centrifuged at 13,000 rpm at 4 °C for 10 min, and the platelet-poor plasma supernatant was stored frozen at −80 °C until analyses.

#### 2.1.4. Plasma EV Isolation

EVs were isolated from plasma using size exclusion chromatography (SEC) preceded by filtration/concentration steps as previously reported [19]. Plasma samples (150 µL) were aliquoted into 1 mL microcentrifuge tubes and centrifuged at 2500× *g* for 10 min at 25 °C. Supernatant was collected and filtered/concentrated through a 100 KDa filter (#UFC510096, Millipore Sigma, Burlington, MA, USA) by centrifugation at 14,000× *g* for 5 min at 25 °C. A concentrated plasma sample was collected by inverting the filter into a clean microcentrifuge tube and centrifuging at 1000× *g* for 2 min. The recovered volume of concentrated plasma samples varied between 55 and 95 μL. These volumes were adjusted to a final volume of 150 μL using 0.1 μm filtered 1× PBS and loaded on the SEC columns. Samples were fractionated using 70 nM qEV single columns (SP2, Izon Science, Medford, MA, USA) using an automated fraction collector in 200 μL fractions (Izon Science, Medford, MA, USA). The first three fractions that contain most of the EVs were combined, frozen at −80 °C and lyophilized. Samples were then resuspended in 75 μL 1× PBS and stored at −80 °C until further use.

#### 2.1.5. Plasma EV Characterization

Nanoparticle Tracking Analysis (NTA) was used to determine the concentration and size distribution of EVs isolated from plasma samples. NTA was performed using a NanoSight NS300 (Malvern Panalytical, Malvern, Worcestershire, UK) equipped with a high-sensitivity sCMOS camera, an automatic syringe pump and a 532 nm laser. Then, 5 µL EV samples resuspended in 1× PBS were thawed on ice and further diluted to 1 mL with 1× PBS prior to injection. The camera and detection settings are provided in Supplementary Table S9. Videos were captured and processed using NTA 3.3 Dev Build 3.3.104 (Malvern, Worcestershire, UK) with 3 videos of 60 s per measurement. All samples were analyzed with automatic syringe movement set to 100. The number of EV particles/mL (concentration) and total EV particles/sample (yield) were calculated based on dilution applied and the total working solution obtained. Our NTA results are consistent with previous reports for a similar size distribution and number of EVs per mL of plasma, strongly suggesting EV enrichment from fractionated plasma samples for metabolomics characterization using high-resolution mass spectrometry (Supplementary Figure S1A). We observed that the EV preparations tested positive for known EV markers, including cluster of differentiation 81 (CD81), cluster of differentiation 63 (CD63), intracellular adhesion molecule (ICAM), ALG-2-interacting Protein X (ALIX), tumor susceptibility gene 101 (TSG101), Annexin5, flotilin1 (Flot1) and epithelial cell adhesion molecule (EpCAM) (Supplementary Figure S1B).

EV immunoblot array: To characterize the isolated EVs and confirm the isolation of bonafide EVs, we performed the Exo-check Antibody Array (System Biosciences, Palo Alto, CA, USA, #EXORAY210A) and examined the expression of classical EV-specific protein markers (TSG101, CD63, CD81, EpCAM, ALIX, Annexin5, Flotilin1, ICAM). EVs resuspended in 1× PBS were thawed on ice, and total protein was quantified by a bicinchoninic acid assay. EV samples consisting about 30 µg of protein were aliquoted. Immunoblots were processed and developed according to the manufacturer’s protocol. A detailed characterization of the EV preparations was performed in accordance with MISEV guidelines. The examined EV-specific protein markers included the transmembrane proteins such as CD63 and CD81 as well as cytosolic proteins like TSG101 and ALIX in the plasma EVs.

### 2.2. Mass Spectrometry Protocols

#### 2.2.1. Untargeted Plasma and Plasma EV Metabolomics and Lipidomics Using UPLC-QToF-MS

Sample preparation for plasma: Plasma samples were taken out of storage at −80 °C and thawed on ice. For extraction, 75 μL of chilled extraction buffer that was prepared by mixing water:methanol:isopropanol (35:25:40) and containing internal standards (debrisoquine and 4-nitrobenzoic acid) was added to 25 µL of plasma sample. The samples were vortexed and incubated on ice for 20 min. Next 100 μL of chilled acetonitrile was added, vortexed and incubated at −20 °C for 20 minutes. Finally, the samples were centrifuged at 13,000 rpm for 20 minutes at 4 °C. The supernatants were transferred to MS vials for LC-MS data acquisition. Then, 20 µL from each sample was mixed to generate a pooled QC sample.

Sample preparation for plasma EV: The EV samples (45 µL resuspended in 1× PBS) stored at −80 °C were thawed, placed on dry ice for 30 s and heat shocked by plunging them into a 37 °C water bath for 90 s. This cycle was repeated two more times followed by sonication for 1 min and then incubation on ice for 20 min. Next, 135 μL of chilled extraction buffer, prepared by mixing water:methanol:isopropanol (35:25:40) containing internal standards (debrisoquine and 4-nitrobenzoic acid), was added. Samples were then vortexed and kept on ice for 20 min. Next, 135 μL of chilled acetonitrile was added and samples were vortexed again for 30 s followed by incubation at −20 °C for 20 min. Finally, samples were centrifuged at 13,000× *g* for 20 min at 4 °C and the supernatants were transferred to MS vials for LC-MS data acquisition. Then, 20 µL from each sample was mixed to generate a pooled QC sample.

Data acquisition: The sample queue was randomized to avoid bias. For metabolomics data acquisition, each sample (1 μL) was injected to a 130Å, 1.7 μm, 2.1 mm  ×  50 mm Acquity BEH C18 column (Waters Corporation, Milford, MA, USA) using an Acquity UPLC system connected to an electrospray ion source coupled with a quadrupole time-of-flight mass spectrometer (ESI-Q-TOF, Xevo-G2S, Waters Corporation, Milford, MA, USA) operating in positive and negative ionization mode. The gradient mobile phases consisted of 100% water with 0.1% formic acid (solvent A), 100% acetonitrile with 0.1% formic acid (solvent B), 100% isopropanol with 0.1% formic acid (solvent C). Each sample injection was run for 13 min at a flow rate for 400 µL/min. The LC gradient conditions with a ramp curve of 6 at each step were as follows: Initial—98% A, 2% B till 0.5 min; 4.0 min—40% A, 60% B; 8.0 min—2% A, 98% B till 9.0 min; 9.5 min—11.8% B, 88.2% C till 11 min; 11.5 min—50% A, 50% B, 12.0 min—98% A, 2% B till 13 min. For lipidomics data acquisition, each sample (1 μL) was injected to a 1.7 μm, 2.1 mm  ×  100 mm Acquity CSH C18 column (Waters Corporation, Milford, MA, USA) using an Acquity UPLC system connected to an electrospray ion source coupled with a quadrupole time-of-flight mass spectrometer (ESI-Q-TOF, Xevo-G2S, Waters Corporation, Milford, MA, USA) operating in positive and negative ionization mode. The gradient mobile phase consisted of 100% water with 0.1% formic acid and 10 mm ammonium formate (solvent A), 100% acetonitrile with 0.1% formic acid (solvent B), 100% isopropanol with 0.1% formic acid (solvent C). Each sample injection was run for 11 min at a flow rate for 450 µL/min. The LC gradient conditions with a ramp curve of 6 at each step were as follows: Initial—30% A, 34% B, 36% C till 0.5 min; 8 min—0% A, 10% B, 90% C till 8.5 min; 9.0 min—30% A, 34% B, 36% C till 11 min.

MS parameters: In the MS method, positive mode had a capillary voltage of 3.0 kV, a sampling cone voltage of 30 V, and a source offset of 80 V. Negative mode had a capillary voltage of 2.0 kV, a sampling cone voltage of 40 V (30 V for lipidomics), and a source offset of 80 V. The desolvation gas flow was 1000 L/h and the temperature was set to 500 °C. The cone gas flow was 25 L/h and the source temperature was 120 °C. The data were acquired in the sensitivity MS Mode with a scan time of 1.000 s, and inter-scan delay at 0.014 s.

The data were acquired in centroid TOF-MS mode over a mass range from 50 to 1200 *m*/*z*. Accurate mass was maintained by infusing Leucine Enkephalin (556.2771 *m*/*z*) in 50% aqueous acetonitrile (0.5 ng/mL) at a rate of 20 µL/min via the Lockspray interface for real-time mass correction, every 10 s. Before and after samples run, a mixture of six standards (acetaminophen: *m*/*z* 152.0712 [M+H]^+^/150.0555 [M−H]^−^, sulfaguanidine: *m*/*z* 215.0603 [M+H]^+^/213.0446 [M−H]^−^, sulfadimethoxine: *m*/*z* 311.0814 [M+H]^+^/309.0658 [M−H]^−^, Val-Tyr-Val: *m*/*z* 380.2185 [M+H]^+^/378.2029 [M−H]^−^, terfenadine: *m*/*z* 472.3216 [M+H]^+^ and leucine-enkephalin: *m*/*z* 556.2771 [M+H]^+^/554.2615 [M−H]^−^) were run to ensure mass accuracy during data acquisition. Several measures were used to ensure the high quality and reproducibility of LC-MS data. The column was conditioned using pooled QC samples, which were injected periodically through the batch to monitor mass accuracy, shifts in retention time and signal intensities as measures of reproducibility. The overlap of QC sample chromatograms (base peak intensity) shows minimal shifts in retention time and consistency in peak intensities throughout the acquisition. Pooled QC samples injected routinely were also used to assess and correct for any analytical variance.

UPLC-QToF-MS data pre-processing: The MS generated raw data files were first converted into NetCDF files for pre-processing using Waters MassLynx Databridge Software within MassLynx v4.1 (Waters Corporation, Milform, MA, USA). XCMS was used for pre-processing the data files while the Isotopologue Parameter Optimization (IPO) package Version 1.14.0 (open-source bioinformatics software package developed by researchers at JOANNEUM RESEARCH Forschungsgesellschaft mbH, Graz, Styria, Austria) was used for XCMS parameter optimization [28]. Normalization was performed with internal standards in both positive and negative mode data. The features with more than 20% missing value were also filtered out and the features with less than 20% of missing value were imputed by half of the minimum positive value in the original data. The features with more than 25% of coefficient of variation (CV) were also filtered out. The remaining high-quality features were normalized by QC-RLSC [29]. R package was used to perform multivariate analysis [30]. Statistically significant *m*/*z*s with FDR adjusted *p*-value < 0.05 were run on the UPLC-QToF instrument (Waters Corporation, Milford, MA, USA) in the MS/MS mode. The MS/MS raw data files were converted to NetCDF files using Waters MassLynx Databridge Software, then further converted to MSP file format using an in-house R package. Thereafter, for accurate mass-based putative identification of metabolites, we performed database search by matching MS/MS spectra to entries in the NIST 2017 MS/MS database and by applying the online version of CEU Mass Mediator (CMM) (an open-access bioinformatics web tool, developed and maintained by the Centre for Metabolomics and Bioanalysis (CEMBIO) at Universidad San Pablo-CEU, Madrid, Madrid, Spain) which integrated from METLIN, Human Metabolome Database (HMDB) and LIPID MAPS with a ppm error of less than 10.

#### 2.2.2. Targeted Metabolomics and Lipidomics Using Sciex 5500 QTRAP

We employed in-house-developed targeted multiple reaction monitoring (MRM)-based quantitative lipidomics and metabolomics analytical methodologies. For this purpose, all LC-MS-grade solvents, including acetonitrile and water, were purchased from Fisher Optima-grade, Fisher Scientific. High-purity formic acid (99%) was purchased from Thermo-Scientific (Waltham, MA, USA). Debrisoquine and 4-nitrobenzoic acid were purchased from Sigma-Aldrich. EquiSPLASH^®^ LIPIDOMIX^®^ quantitative mass spec internal standard and 15:0–18:1-d7-PA, C15 Ceramide-d7 (d18:1-d7/15:0), and 18:1 Chol (D7) ester were purchased from Avanti Polar Lipids (Alabaster, AL, USA). Internal standards for free fatty acids (FFAs), dihydroceramides (DCER), hexosylceramides (HCERs), and lactosylceramides (LCERs) were purchased from Sciex as Lipidyzer platform kits. All samples were randomized, processed and analyzed using standardized procedures to minimize potential technical variation. Details of the sample preparation, LC-MS conditions, data acquisition, data pre-processing, and statistical analyses have been detailed below.

Plasma sample preparation: Polar plasma metabolites and lipids were extracted using a protein precipitation-based protocol optimized for LC–MS/MS analysis. For this purpose, 25 µL of each plasma sample was mixed with 125 µL of chilled isopropanol containing internal standards. The samples were vortexed for 1 min and kept on ice for 30 min, then incubated at −20 °C for 2 h to allow protein precipitation. Subsequently, the samples were centrifuged at 13,000 rpm for 20 min at 4 °C. The resulting supernatant was transferred to MS vials for LC–MS analysis. To generate a pooled quality control (QC) sample, 20 µL of each prepared sample was combined.

Plasma EV sample preparation: Polar EV metabolites and lipids were extracted using a protein precipitation-based protocol optimized for LC–MS/MS analysis. For this purpose, 15 µL of each EV sample was mixed with 40 µL of a water/methanol/isopropanol mixture (35:25:40, *v*/*v*/*v*). The sample tubes were subjected to three freeze–thaw cycles by alternating immersion in dry ice for 30 s and a 37 °C water bath for 90 s, followed by sonication for 1 min. Subsequently, 100 µL of chilled isopropanol containing internal standards was added to each sample. The samples were vortexed for 1 min and kept on ice for 30 min, followed by incubation at −20 °C for 2 h to facilitate protein precipitation. The samples were then centrifuged at 13,000 rpm for 20 min at 4 °C, and the resulting supernatant was transferred to MS vials for LC–MS analysis. To generate a pooled QC sample, 20 µL of each prepared sample was combined.

Targeted metabolomics acquisition: Targeted metabolomics analysis was performed using a QTRAP 5500 LC–MS/MS system (SCIEX, Marlborough, MA, USA) operated in scheduled multiple reaction monitoring (MRM) mode with polarity switching to enable detection in both positive and negative ion modes. The targeted assay was developed in-house and enabled quantification of 270 endogenous metabolites spanning multiple biochemical pathways. Chromatographic separation was achieved on a Kinetex F5 column (2.6 µm, 100 Å, 100 × 2.1 mm; Phenomenex, Torrance, CA, USA) maintained at 30 °C, with a flow rate of 0.200 mL/min. A 5 µL aliquot of each processed sample was injected using a SIL-30AC autosampler (Shimadzu, Kyoto, Japan), which was coupled to an LC-30AD solvent delivery unit and CBM-20A communication module (Shimadzu, Kyoto, Japan), with the autosampler temperature maintained at 15 °C and a 2 s needle wash using a 1:1:1:1 water/isopropanol/methanol/acetonitrile rinse solvent. The mobile phases consisted of water with 0.1% formic acid (A) and acetonitrile with 0.1% formic acid (B). The gradient was programmed as follows: 0–2.1 min, 100% A; 2.1–14.0 min, linear decrease to 5% A; 14.0–16.0 min, 5% A and 95% B; 16.1–20.0 min, return to 100% A, with a total run (stop) time of 21.0 min and a maximum pressure limit of 15,000 psi. The mass spectrometer was operated with a turbo spray ion source at 400 °C, using curtain gas at 35 psi, heater gas at 60 psi, ion source gas 1 at 60 psi, ion source gas 2 at 70 psi, and nebulizing gas at 50 psi. The capillary voltage was set to +5500 V in positive mode and −4500 V in negative mode, with medium collision-activated dissociation (CAD) gas and an entrance potential of 10 V.

Targeted lipidomics acquisition: Targeted lipidomics analysis was performed using the same QTRAP 5500 LC–MS/MS system configured with class-specific MRM transitions to enable comprehensive coverage and quantification of major lipid subclasses, including phospholipids (phosphatidylcholines, lysophosphatidylcholines, phosphatidic acids, lysophosphatidic acids, phosphatidylethanolamines, lysophosphatidylethanolamines, phosphatidylglycerols, phosphatidylserines, phosphatidylinositols, and lysophosphatidylinositols), glycerolipids (monoacylglycerols, diacylglycerols, and triacylglycerols), sphingolipids (sphingomyelins, ceramides, hexosylceramides, dihydroceramides, and lactosylceramides), free fatty acids, cholesterol esters, and acylcarnitines. Chromatographic separation was achieved on an XBridge BEH Amide column (100 × 4.6 mm, 3.5 µm; Waters, MA, USA) maintained at 30 °C. The mobile phases consisted of 10 mM ammonium acetate in 95:5 acetonitrile/water (A) and 10 mM ammonium acetate in 50:50 acetonitrile/water (B). The LC gradient was run at a flow rate of 0.2–0.7 mL/min as follows: 0–3.0 min, 100% A; 3.0–3.05 min, 99.9% A/0.1% B; 3.05–3.10 min, 99.8% A/0.2% B (flow increased to 0.7 mL/min); 3.10–6.0 min, ramp to 94% A/6% B; 6.0–10.0 min, ramp to 75% A/25% B; 10.0–11.0 min, 2% A/98% B; 11.0–13.0 min, 0% A/100% B; 13.0–18.6 min, 0% A/100% B at 0.7 mL/min; 18.6–18.8 min, return to 100% A while reducing flow to 0.2 mL/min; 18.8–22.0 min, 100% A at 0.2 mL/min, with a stop time of 23.0 min and a maximum pressure of 18,000 psi. A 2 µL aliquot of each sample was injected using an autosampler maintained at 15 °C, with a 2 s needle wash using a 1:1:1:1 water/isopropanol/methanol/acetonitrile rinse solvent. The mass spectrometer was operated with a turbo spray ion source at 550 °C, using curtain gas at 30 psi, heater gas at 60 psi, ion source gas 1 at 50 psi, ion source gas 2 at 60 psi, and medium collision gas. The capillary voltage was set to +5500 V in positive mode and −4500 V in negative mode.

Quality Control and Instrument Performance Monitoring: To monitor analytical reproducibility and instrument stability, a pooled quality control (QC) sample was prepared by combining 10 µL from each processed plasma/EV sample. All samples were maintained at low temperatures during preparation to minimize metabolite degradation and prevent artifactual changes. Stringent quality control procedures were implemented to ensure high analytical reproducibility and data robustness. Prior to batch acquisition, the chromatographic column was conditioned using 5 repeated injections of pooled QC samples to stabilize retention times and signal intensities. During the analytical run, pooled QC samples were injected after every 10 study samples to monitor instrumental drift, retention time stability, and signal variation. Blank solvent injections were included after every 10 samples, both before and after QC injections, to evaluate potential sample carry-over and background contamination. Additionally, a NIST standard reference plasma sample was analyzed after every 20 injections to monitor instrument performance and inter-batch consistency. Analytical signal drift across the batch was corrected using quality control-based robust locally estimated scatterplot smoothing (QC-RLSC).

Data processing and statistical analysis: The abundance measurement for metabolites and lipids were derived as intensity units that were initially normalized to internal standards and processed using MultiQuant 3.0.3 (SCIEX). The data were pre-processed using a signal/noise ratio >20:1 and retention time (RT) tolerance of 5 s, after manually checking of metabolites peak by experts to find the reliable features. We also use 20% of missing values in each feature as filter-out criteria. Missing values were imputed by half of the minimum positive value in the original data. Thereafter, we used 20% of the coefficient of variation (CV) as our filter criteria to remove any possible noises before data normalization. Analytical drifts (if any) were corrected by quality control-based robust LOESS signal correction (QC-RLSC). All the analyses were performed in MetaboAnalyst (V5.0) and R (v 4.0.3). The normalized LC-MS data was log-transformed and Pareto-scaled. For the 185 samples in the study set, the level of differential expression for each metabolite was calculated using an unpaired *t*-test, comparing sham vs. radiation, and radiation vs. radiation plus APC-treated samples, constrained by the FDR-corrected *p*-value < 0.05. MetaboAnalyst (V5.0) was used to evaluate the metabolic pathways affected by radiation exposure longitudinally and radioprotective alleviation by APC treatments.

## 3. Results

### 3.1. Whole Plasma and Plasma-Derived EVs Have Distinct Metabolite and Lipid Profiles

**Figure 1:**
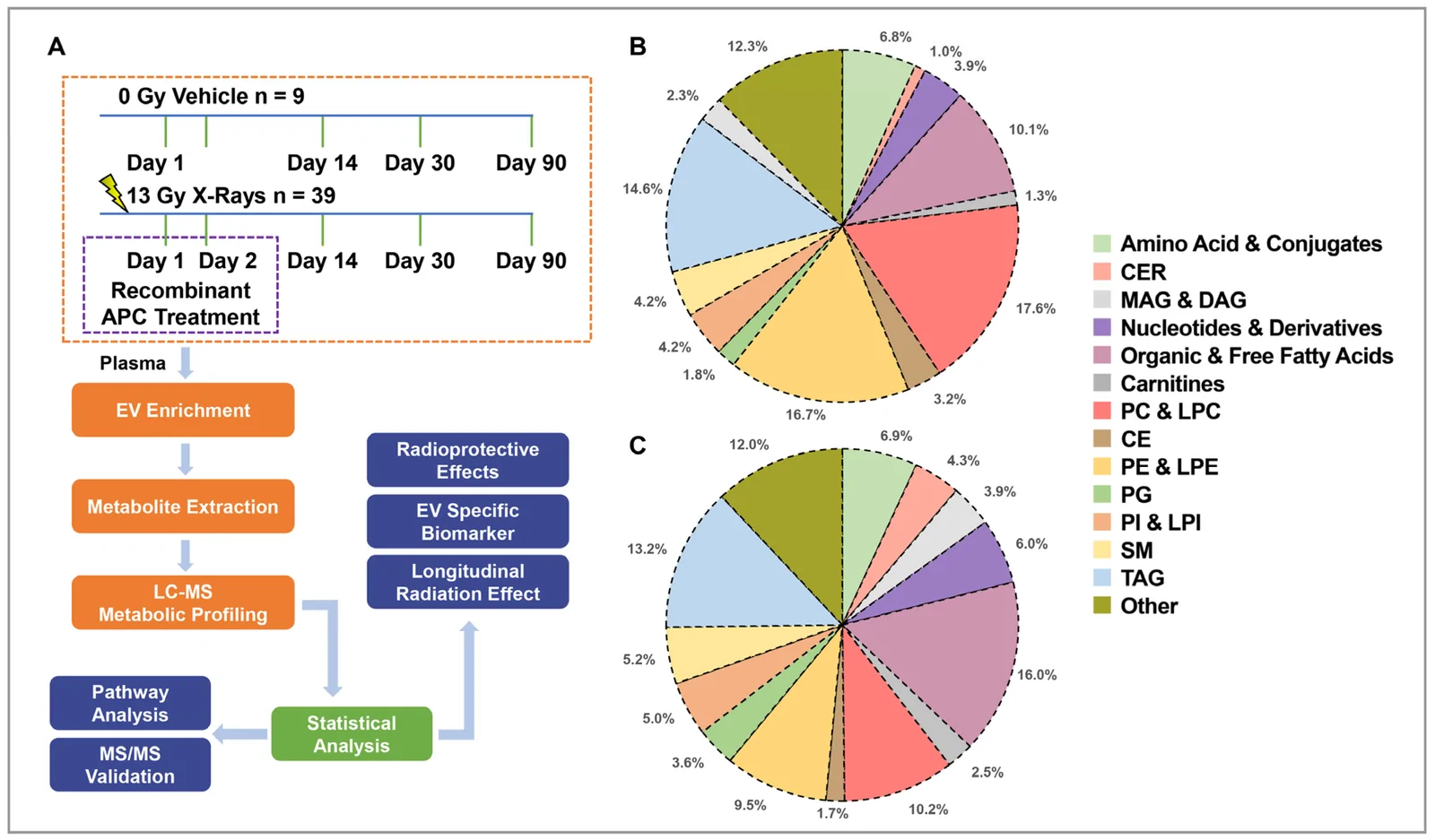
Panel (**A**). Experimental and analytical design of the study. Plasma samples were collected from WAG/RijCmcr female rats at days 1, 14, 30 and 90 after 13 Gy leg-out partial body irradiation (PBI) or sham treatment and processed for LC-MS-based metabolomic and lipidomic analyses. Panels (**B**,**C**). Pie chart showing differential class distribution of metabolites in plasma (**B**) and plasma-derived EVs (**C**) upon irradiation. These pie charts highlight the shifts in metabolite classes, offering a visual representation of the biochemical changes occurring post-irradiation.

In this study, adult female WAG/RijCmcr rats (*n* = 48) were subjected to leg-out PBI (13.0 Gy X-rays) (*n* = 39) or sham exposure (*n* = 9) to evaluate IR-induced alterations in the metabolomic and lipidomic composition of whole plasma and plasma-derived EV-enriched fractions. A sub-cohort of 30 rats received two doses (24 and 48 h post-irradiation) of one of three recombinant APC variants: rat WT APC (*n* = 10), rat 3K3A-APC (*n* = 10), or human WT APC (*n* = 10), to assess their potential as IR mitigators. Plasma samples were collected longitudinally at days 1, 14, 30, and 90 and analyzed by LC-MS-based global and targeted metabolomics and lipidomics (Figure 1A). Plasma and EV-enriched fractions exhibited distinct patterns of biochemical remodeling following IR exposure; in particular, Figure 1B,C summarize the class-level composition of metabolites detected in plasma and plasma-derived EVs, respectively, illustrating IR-induced shifts in metabolite class distributions and demonstrating enrichment of specific lipid classes in EVs relative to bulk plasma. These descriptive profiles establish a baseline compositional framework and justify our focus on EV cargo as a potentially more sensitive matrix for detecting IR-induced lipid perturbations in the subsequent biomarker analyses. Although there was a substantial overlap in overall metabolite coverage, the relative distribution of lipid classes differed markedly: plasma exhibited higher prevalence of several phospholipid classes, whereas EVs were enriched in sphingolipids, glycerolipids, free fatty acids (FFAs), and acylcarnitines. These EV-enriched lipid classes are particularly sensitive to redox imbalance and lipid peroxidation [31,32,33]. Consistent with this, EVs and plasma displayed distinct yet complementary metabolic signatures following IR. While amino acids, amino acid conjugates, and several other small molecules were similarly represented in both matrices, EVs showed relatively higher levels of organic acids and nucleotide/nucleoside/nucleobase species and their conjugates. Together, these observations support the notion that EVs may serve as active carriers of redox-relevant metabolites and intermediates of nucleic acid turnover, potentially contributing to the systemic propagation or modulation of IR-induced oxidative damage. Consistent with this, the EV-enriched fractions exhibited a distinct and characteristic lipid profile compared with bulk plasma (Supplementary Figure S2).

### 3.2. IR-Induced Metabolic Changes in Plasma and EVs

**Figure 2:**
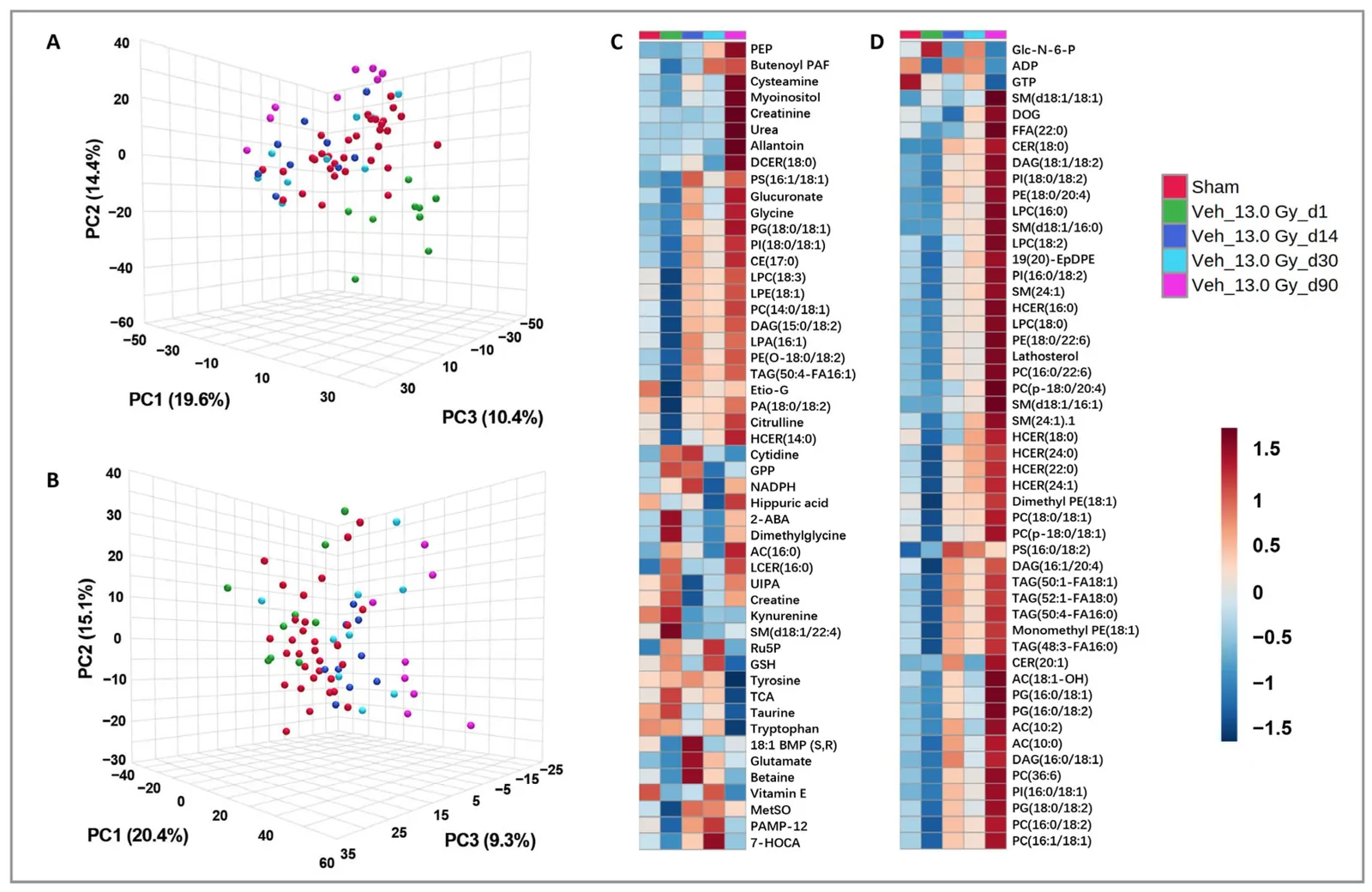
Principal component analysis (PCA) and heatmap visualization of longitudinal metabolic changes in plasma and plasma-derived EVs. Three-dimensional PCA score plots of plasma (**A**) and EV (**B**) metabolomes at days 1, 14, 30, and 90 post-irradiation. PCA was performed as an unsupervised, exploratory analysis; PC1 and PC2 explain 19.6% and 14.4% of the variance in plasma, and 20.4% and 15.1% in EVs, respectively. Heatmaps showing normalized abundances of the top 50 significantly altered metabolites in plasma (**C**) and EVs (**D**). Rows represent individual metabolites, and columns represent group–time point combinations (sham, IR at days 1, 14, 30, and 90 post-irradiations). In Panels (**A**,**B**), colors indicate treatment group and time point: red, sham (0 Gy); green, 13.0 Gy day 1; blue, 13.0 Gy day 14; cyan, 13.0 Gy day 30; magenta, 13.0 Gy day 90 post-irradiation.

To assess the utility of EVs as a complementary matrix for discovering novel biomarkers, initially, we evaluated IR-induced changes in plasma and EV metabolomes using unsupervised PCA (Figure 2A,B) and examined the differential distribution of metabolites and lipid classes between the two matrices. We leveraged in-house developed targeted MRM-based quantitative lipidomics and metabolomics analytical methodologies. Rigorous data processing and quality control (Supplementary Figure S3) of plasma yielded 196 metabolites and 541 lipids, while profiling of the plasma-EV fraction provided 195 metabolites and 372 lipids suitable for quantitative analysis. Complementary global LC-MS profiling with Isotopologue Parameter Optimization (IPO)-based XCMS deconvolution detected 5571 and 4952 features in plasma metabolomics and lipidomics modes, respectively, and 3483 and 3478 features in EV metabolomics and lipidomics, underscoring the broad impact of IR on both the circulating and EV-encapsulated small-molecule repertoire. Following data normalization and log transformation, we performed multivariate analyses that revealed clear separation between X-ray exposed and sham-irradiated rats, with distinct clustering at days 1 and 90 post-irradiation in both plasma and EVs, consistent with acute and delayed phases of IR-induced oxidative and metabolic stress. Using a fold-change threshold ≥2.0 and FDR-adjusted *p*-value ≤ 0.05, volcano plots (Supplementary Figure S4, Supplementary Tables S1–S4) highlighted a pronounced acute impact at day 1, followed by a relative attenuation of dysregulation at days 14 and 30. By three months, however, a renewed and enhanced dysregulation emerged, consistent with DEARE and indicative of persistent, progressive metabolic perturbations. Significantly altered features were putatively identified by accurate-mass matching (ppm < 10) using CEU Mass Mediator (integrating METLIN, HMDB, and LIPID MAPS), followed by MS/MS-based fragmentation pattern matching against the NIST 2017 library, a widely accepted pipeline for untargeted feature validation [34,35]. In total, 83 plasma and 33 EV features were putatively annotated (Supplementary Tables S5 and S6). The longitudinal heatmaps in Figure 2C,D depict the top 50 altered metabolites in plasma and EVs, respectively, highlighting matrix-specific trajectories of lipids and metabolites closely linked to IR-induced stress and its partial, time-dependent mitigation likely by endogenous defense mechanisms.

IR-induced alterations in the plasma lipidome were dominated by changes in phospholipids and glycerolipids, while sphingolipids, acylcarnitines, and sterol lipids showed relatively modest changes. Most lipid classes, except for sphingomyelins (SMs) and acylcarnitines, showed decreased levels at day 1 post-irradiation, followed by partial recovery by day 14. In contrast, SMs were elevated at day 1 but declined by day 90, a pattern compatible with IR-driven stress activating acid sphingomyelinase and promoting SM hydrolysis to ceramides [36]. Fatty acyls, including polyunsaturated fatty acids (PUFAs) and oxylipins—key substrates and products of lipid peroxidation—also showed reduced levels at day 90, suggesting sustained oxidative stress-induced changes.

Similarly to plasma, the EV lipidome showed dysregulation of glycerolipids, phospholipids, sphingolipids, and acylcarnitines. However, while plasma lipid perturbations were more prominent during the acute phase (up to day 14, consistent with ARS), most EV lipidomic changes emerged at day 90, suggestive of DEARE and indicating delayed, ongoing lipid abnormalities. EVs also exhibited a higher number of altered SMs and hexosylceramides (HCERs). Strikingly, SMs in EVs showed the opposite temporal pattern to plasma, with downregulation at day 1 and upregulation at day 90. In addition, specific ceramides (18:0 and 20:1) and FFA 22:0 were selectively upregulated in EVs at day 90, and acylcarnitines, often linked to mitochondrial β oxidation and redox status, were increased in EVs but not in plasma at this time point. This pattern suggests that EVs preferentially accumulate and export lipids linked to mitochondrial β-oxidation and ceramide signaling during the delayed phase of the response to IR.

### 3.3. IR-Induced Dysregulation in PLASMA and EV Metabolic Pathways

**Figure 3:**
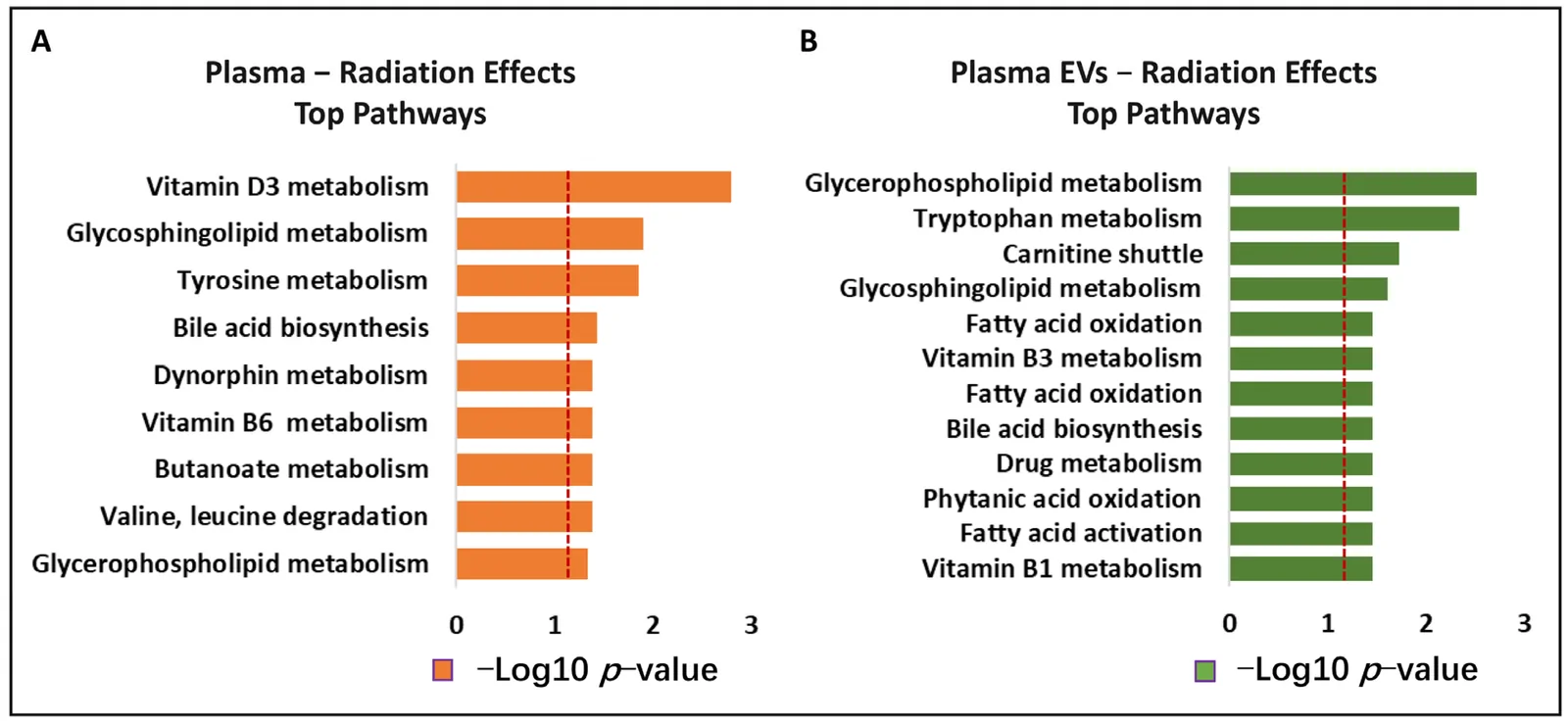
Mummichog pathway analysis of top pathways dysregulated by IR at day 30. Panel (**A**) shows plasma and Panel (**B**) shows plasma-derived EVs, highlighting pathways most affected by IR in each compartment. Pathway enrichment was performed independently at all four post-irradiation time points (days 1, 14, 30, and 90) using Mummichog with *p* < 0.05 as the significance threshold; full pathway lists, and matched feature counts are provided in the Supplementary Tables S7 and S8. The red dashed vertical line in each panel denotes the significance threshold (−Log10 *p* = 1.30, i.e., *p* < 0.05).

Next, we performed Mummichog pathway analysis on dysregulated metabolites (FDR *p*-value < 0.05 and log2 fold change >2 or <0.5) detected in plasma and enriched EVs from animals exposed to 13 Gy PBI (Supplementary Tables S7 and S8). Feature tables containing *m*/*z* values, retention times, and associated *p*-values were exported and analyzed using the Mummichog algorithm [37], with KEGG-based rat metabolic pathway libraries and LC–MS parameters (positive/negative ion mode, high-resolution MS) matched to the acquisition conditions. Briefly, Mummichog maps *m*/*z* features to putative metabolites considering relevant adducts and then projects these onto curated metabolic pathways; enrichment statistics are computed using Fisher’s exact (hypergeometric) test, with additional permutation-based gamma statistics to model a null distribution for pathway level *p* values. Mummichog enrichment was performed independently at all four time points (days 1, 14, 30, and 90) in plasma and EVs (Supplementary Tables S7 and S8). Across the time-course, plasma dysregulation peaked at day 1–14, attenuated by day 30, and re-intensified by day 90, while EV pathway changes were minimal at day 1 and grew progressively to peak at day 90, reflecting delayed, DEARE-associated involvement. Bile acid biosynthesis and glycerophospholipid metabolism were persistently dysregulated across nearly all time points in both matrices, tryptophan and vitamin B1/B3 metabolism were acute phase-restricted, and fatty acid β-oxidation, the carnitine shuttle, and glycosphingolipid/ceramide metabolism emerged specifically from day 30 onward—together defining an acute, persistent, and delayed pathway structure consistent with the transition from ARS to DEARE. At day 30 post-irradiation (Figure 3A,B), plasma and EVs showed overlapping changes in several redox-related pathways, including glycerophospholipid and glycosphingolipid metabolism, fatty acid activation and (peroxisomal) β-oxidation, the carnitine shuttle, bile acid biosynthesis, and tryptophan metabolism. These shared alterations may reflect broader changes in membrane composition and organelle function, which could influence lipid handling and overall cellular stress responses. We also observed IR-induced dysregulation of vitamin B1 (thiamin) and vitamin B3 (nicotinate/nicotinamide) metabolism, which are key regulators of NADH/NADPH generation and NAD^+^-dependent redox and repair pathways.

### 3.4. APC Variants Exert Significant Mitigation of IR Effects

APC modulates gene expression and exerts anti-inflammatory, anti-apoptotic, antithrombotic, and cytoprotective effects, playing a key role in maintaining vascular integrity through stabilization of the endothelial barrier [38,39]. Its cytoprotective signaling depends on binding to endothelial protein C receptor (EPCR) and subsequent activation of protease-activated receptor-1 (PAR-1) [40,41]. One of our primary objectives was to determine the potential alleviation of IR-induced injury in metabolic profiles upon treatment with three recombinant APC variants.

All three variants showed some mitigation of IR-associated metabolic perturbations across the studied time points, as reflected by partial restoration of metabolic homeostasis in APC-treated rats. In plasma, the rat 3K3A-APC variant provided the strongest mitigation at the early post-irradiation time point (day 14), whereas rat WT APC and human WT APC were more effective at the later time point (day 90), suggesting a sustained effect on IR-driven metabolic disturbances.

#### 3.4.1. Rat 3K3A APC Mitigates Early IR-Induced Changes in Plasma and EVs

**Figure 4:**
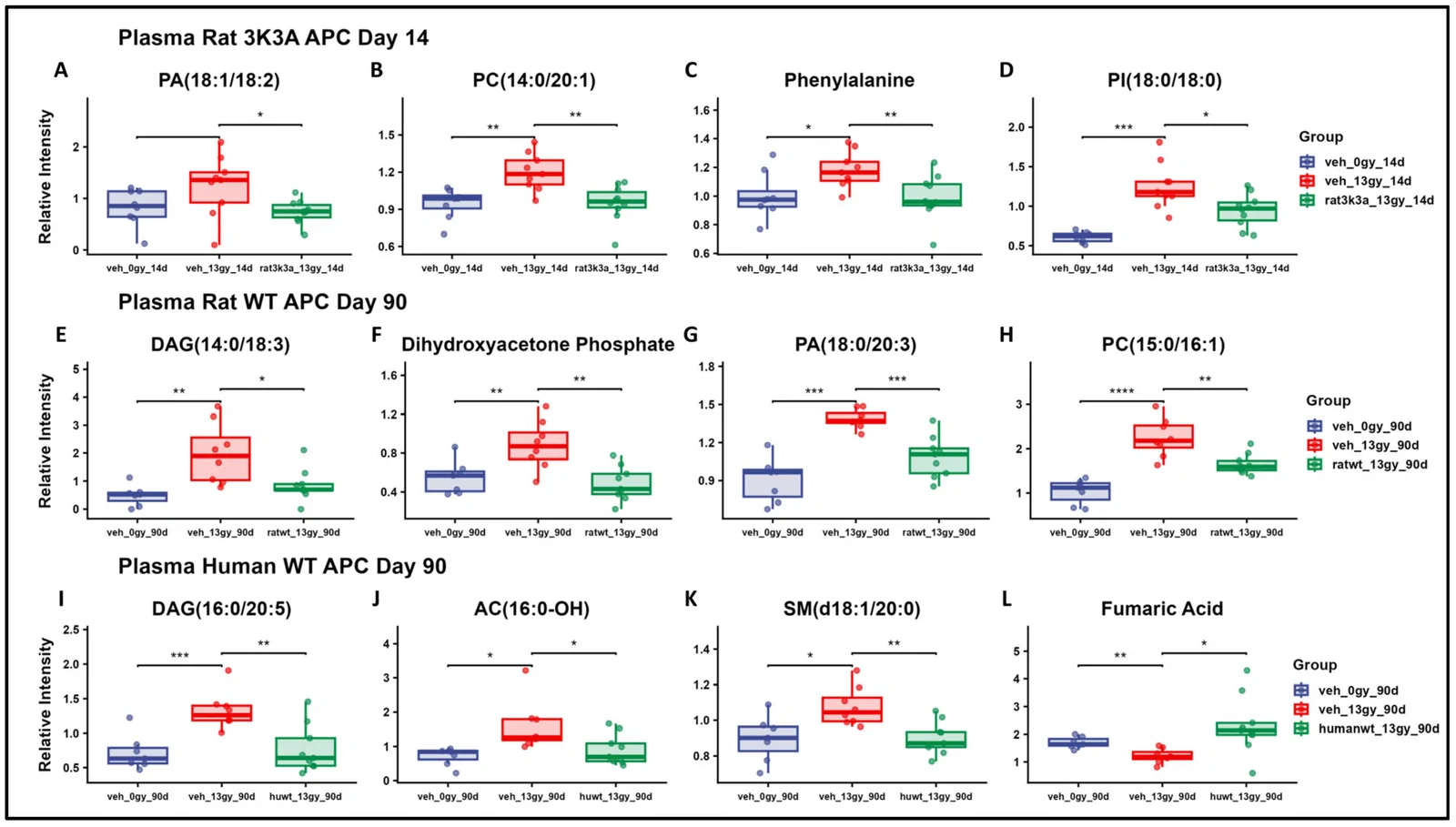
Box plot representation showing reversal of IR-mediated metabolic dysregulation in rat plasma by APC variants. Panels (**A**–**D**): rat 3K3A APC at day 14; Panels (**E**–**H**): rat WT APC at day 90; Panels (**I**–**L**): human WT APC at day 90 (* *p* < 0.05, ** *p* < 0.01, *** *p* < 0.001, **** *p* < 0.0001).

**Figure 5:**
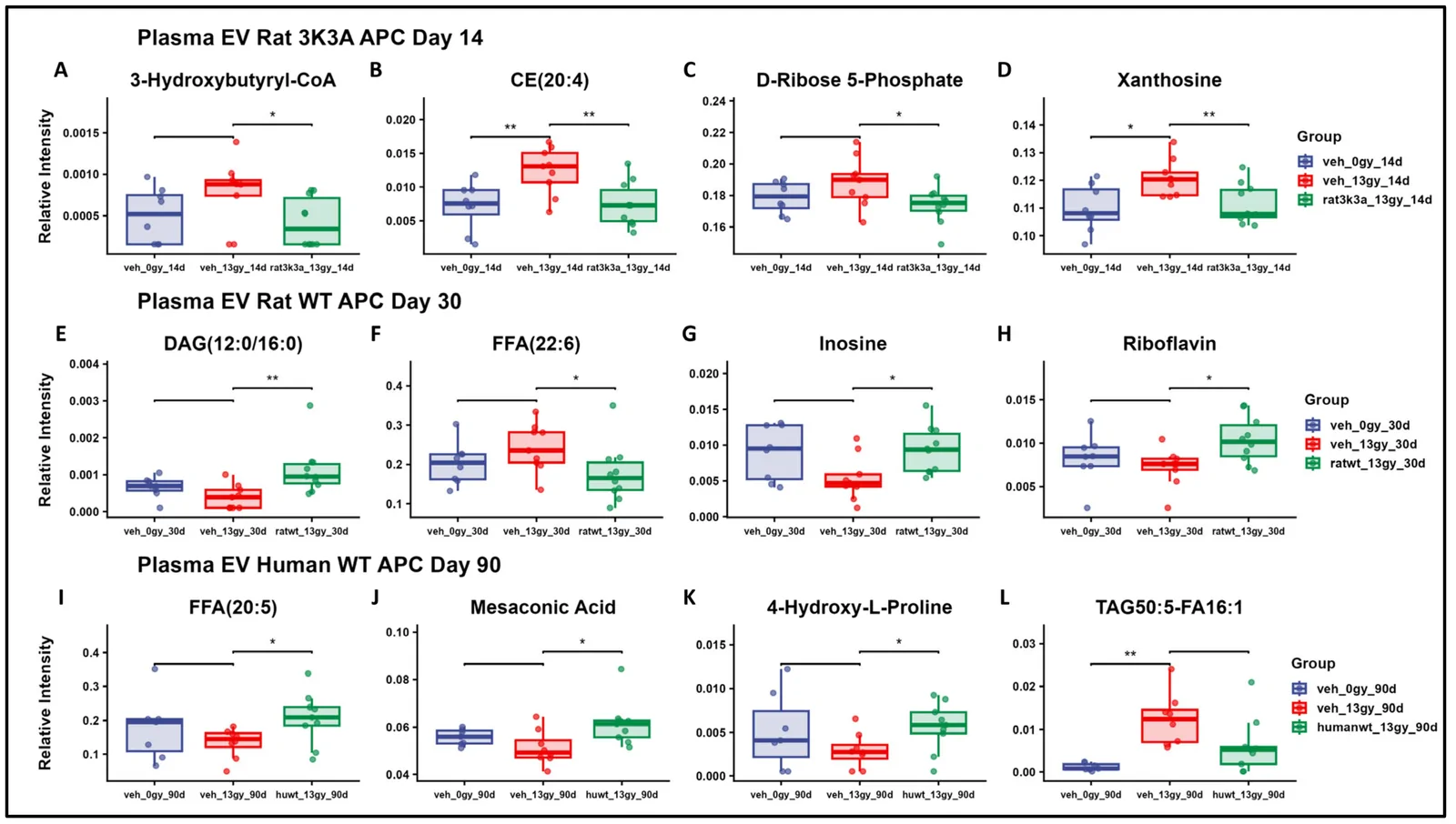
Box plot representation showing reversal of IR-mediated metabolic dysregulation in rat plasma-derived EVs by APC variants. Panels (**A**–**D**): rat 3K3A APC at day 14; Panels (**E**–**H**): rat WT APC at day 30; Panels (**I**–**L**): human WT APC at day 90 (* *p* < 0.05, ** *p* < 0.01).

Most of the IR mitigation effects of rat 3K3A APC were observed at earlier time-points in plasma and EVs. In plasma, IR induced alterations in key membrane lipids—phosphatidic acid (PA), phosphatidylcholine (PC), and phosphatidylinositol (PI) were restored to normal or near-normal levels, consistent with partial correction of oxidative membrane damage and lipid remodeling (Figure 4A–D). In parallel, phenylalanine levels were also returned toward baseline, indicating normalization of systemic amino acid metabolism. In plasma-EVs, metabolites like 3-hydroxybutyryl-Coenzyme A (CoA), cholesteryl ester CE(20:4), ribose-5-phosphate, and xanthosine were restored to normal or near-normal levels (Figure 5A–D). The APC variant rat 3K3A also showed some mitigation of IR effects in the plasma metabolome, and the expression levels of lipids/metabolites like CE(16:0), cis-4-hydroxy-proline, 2-aminobutyric acid, and dihydrofolate were corrected at day 90 (Supplementary Figure S5A–D).

#### 3.4.2. Rat WT APC Ameliorates IR-Induced Dysregulation of Plasma and EV Metabolites

In rats receiving rat WT APC, attenuation of metabolic dysregulation for several metabolites, including 3-hydroxyanthranilic acid, HCER(26:0), PE(14:0/20:4), and phenylalanine, was observed at day 14 in plasma (Supplementary Figure S5E–H). By day 90, recovery of plasma DAG(14:0/18:3), dihydroxyacetone phosphate, PA(18:0/20:3), and PC(15:0/16:1) reflected longer-term stabilization of glycerolipid/glycerophospholipid metabolism (Figure 4E–H). In EVs, by day 30, DAG(12:0/16:0), FFA(22:6), inosine, and riboflavin were restored to normal or near-normal levels (Figure 5E–H).

#### 3.4.3. Human WT APC Attenuates IR-Induced Metabolic Perturbations in a Time-Dependent Manner

In rats treated with human WT APC, by day 90, recovery of DAG (16:0/20:5), acylcarnitine(16:0-OH), SM(18:1/20:0), and fumaric acid in plasma indicated sustained stabilization of mitochondrial β-oxidation, membrane lipid remodeling, and tricarboxylic acid (TCA) cycle (Figure 4I–L). By day 14, partial restoration of glutamate, histidine, phenylalanine, and sarcosine suggested early correction of amino acid and one-carbon metabolism (Supplementary Figure S5I–L). The restoration of FFA(20:5), mesaconic acid, 4-hydroxyproline, and TAG(50:5/16:1) (Figure 5I–L) in EVs at day 90 suggest long-term normalization of omega-3 fatty acid pools and intermediary carbon metabolism.

### 3.5. Metabolic Pathways Mitigated by APC Variants in Plasma and EVs

**Figure 6:**
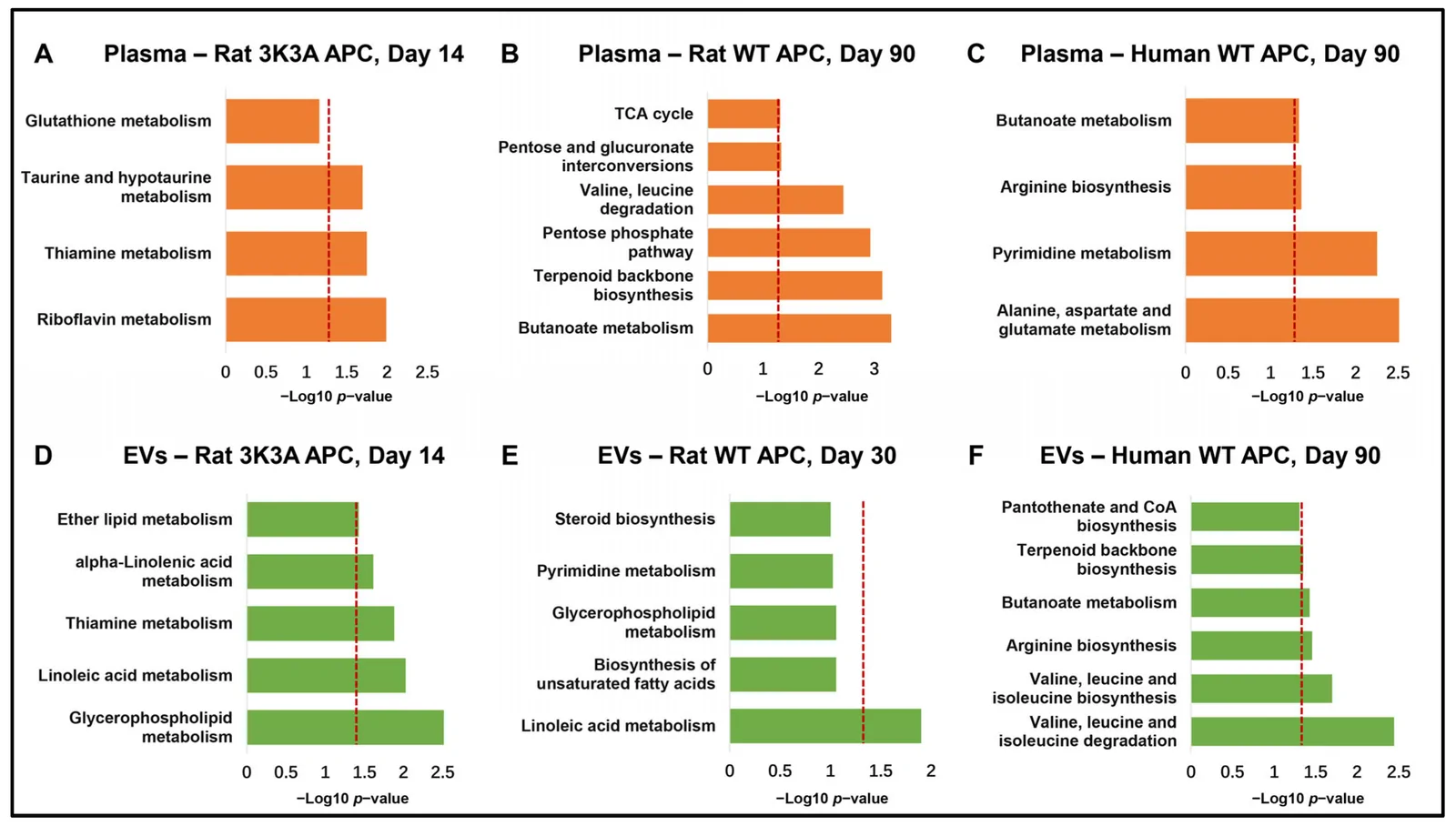
Pathway analysis identified APC variant-specific metabolic perturbations across different time points in plasma and plasma-derived EVs. Metabolic pathway analysis revealed that the 3K3A variant of APC was effective at 14 days, while mitigative effects of rat and human WT APC were most pronounced at 90 days in plasma (**A**–**C**). Panels (**D**–**F**) depict pathway alterations in EVs for 3K3A at day 14, rat WT APC at day 30 and human WT APC at day 90. The red dashed vertical line in each panel denotes the significance threshold (−Log10 *p* = 1.30, i.e., *p* < 0.05).

APC treatment mitigated dysregulation across multiple metabolic pathways in both plasma and EV matrices following IR exposure. At day 14 post-irradiation, rat 3K3A-APC showed the most pronounced early mitigation. In plasma, 3K3A-APC helped alleviate dysregulation of riboflavin, thiamine, taurine and hypotaurine metabolism, as well as glutathione metabolism, consistent with partial preservation of redox-related pathways (Figure 6A). In EVs, 3K3A-APC mitigated changes in glycerophospholipid and ether lipid metabolism, linoleic and α-linolenic acid metabolism, arachidonic acid metabolism, and thiamine metabolism (Figure 6D), indicating early protection of lipid and cofactor pathways within EV cargo.

By day 90, mitigation by human WT APC and rat WT APC became more evident. In plasma, human WT APC helped preserve alanine, aspartate and glutamate metabolism, pyrimidine metabolism, arginine biosynthesis, and butanoate metabolism (Figure 6B), whereas rat WT APC supported butanoate metabolism, terpenoid backbone biosynthesis, the pentose phosphate pathway, valine, leucine and isoleucine degradation, pentose and glucuronate interconversions, and the TCA cycle (Figure 6C). In EVs at day 30, rat WT APC treatment preserved arginine biosynthesis, nicotinate and nicotinamide metabolism, histidine metabolism, pantothenate and CoA biosynthesis, β-alanine metabolism, alanine, aspartate and glutamate metabolism, lysine degradation, and purine metabolism (Figure 6E). EVs from human WT APC-treated rats maintained valine, leucine and isoleucine degradation and biosynthesis, arginine biosynthesis, butanoate metabolism, terpenoid backbone biosynthesis, pantothenate and CoA biosynthesis, and purine metabolism (Figure 6F).

**Figure 7:**
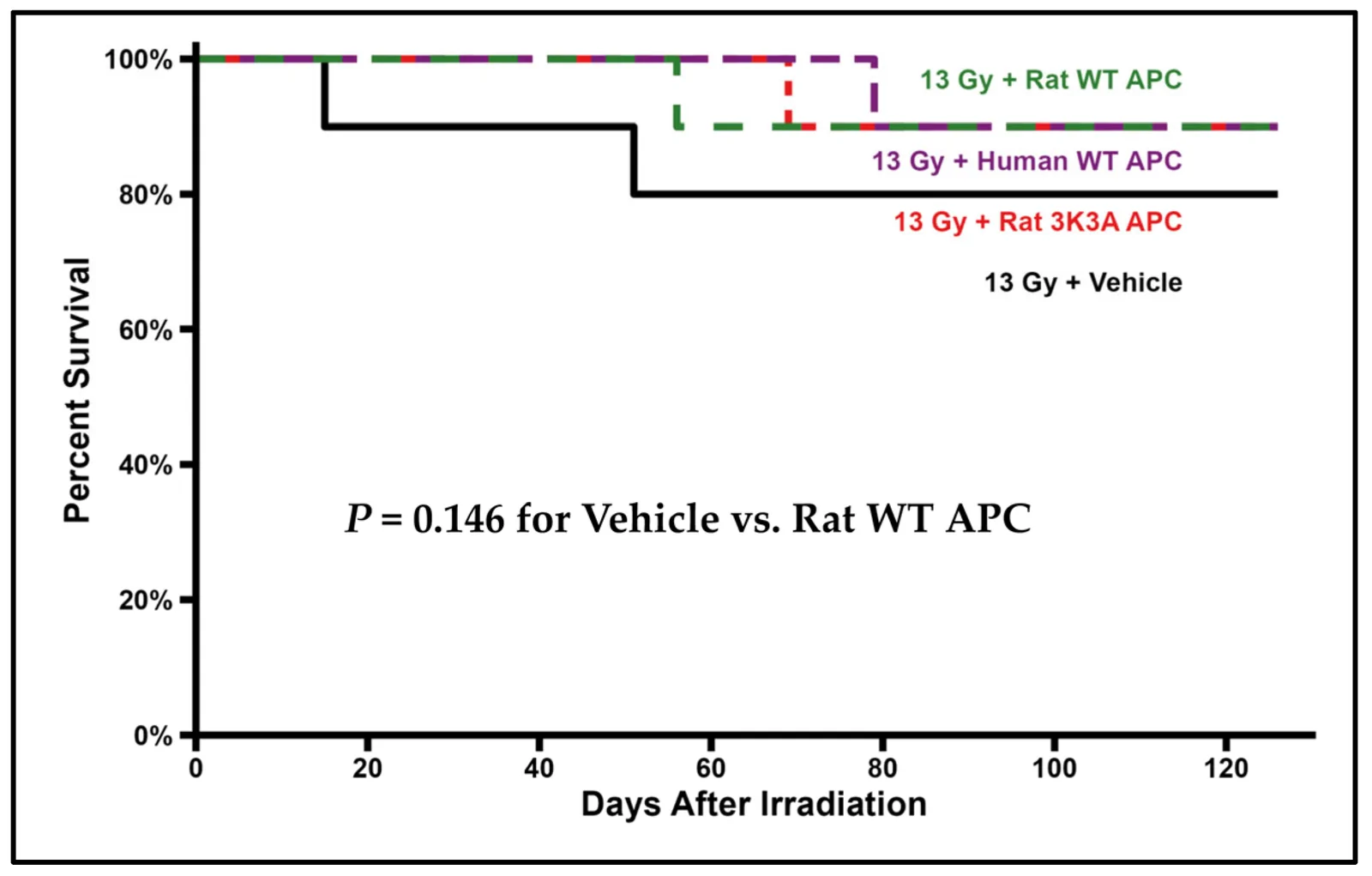
Survival curve after 13 Gy X-ray leg-out PBI and treatments with APC variants in two administrations at 24 and 48 h after irradiation.

Figure 7 shows that leg-out PBI decreased long-term animal survival, with the IR-only group exhibiting roughly 10–30% mortality by day 120. Rats treated with rat WT APC achieved 100% survival and human WT APC and rat 3K3A-APC each were associated with 80% survival at day 120. While these trends suggest a survival benefit of APC treatment, the differences between groups did not reach statistical significance (*p* = 0.146 for vehicle vs. rat WT APC).

## 4. Discussion

Identifying robust biomarkers and key mechanisms of IR injury is vital for developing therapies that can prevent or mitigate ARS and DEARE. Despite an array of studies, there are no FDA-approved biomarker signatures for predicting IR-induced organ injury in the acute or delayed phase. Metabolites and lipids are closely tied to redox balance, membrane integrity, mitochondrial function, and immune responses, making them sensitive early indicators of tissue damage and promising targets for intervention. However, most previous studies have focused on plasma or tissue profiles, paying little attention to how these signatures change from the acute to delayed phases or how EVs may help propagate IR-induced low-abundance stress signals. Moreover, there is a critical need to understand whether candidate radiomitigators, such as APC variants with known cytoprotective properties, can meaningfully modulate these systemic and EV-mediated metabolic changes. In this context, we used longitudinal metabolomic and lipidomic profiling of whole plasma and plasma-derived EVs from rats exposed to a single, high-dose (13 Gy) leg-out X-ray PBI to define IR-responsive pathways across acute and early delayed phases, and to determine the extent to which three recombinant APC variants can normalize these perturbations.

A unique feature of this work is the parallel, longitudinal assessment of both plasma and enriched EV cargo across acute (days 1–14) and early delayed (days 30–90) phases after irradiation. This study allowed us to distinguish shared versus compartment-specific metabolic signatures and to identify EVs as dynamic carriers of IR-induced stress signals. In addition, comparing three APC variants (rat 3K3A, rat wild-type, and human wild-type) provided insight into how structurally distinct cytoprotective agents differentially affect ARS versus DEARE-related pathways. In addition, comparing three APC variants (rat 3K3A, rat wild-type, and human wild-type) provided insight into how structurally distinct cytoprotective agents differentially affect ARS versus DEARE-related pathways. It is noteworthy that rat WT APC did not give exactly the same effects as human WT APC, thereby confirming that species differences in animal-derived reagents and animal subjects merits careful attention.

We observed IR-induced dyslipidemia, which corroborates prior reports that have identified dysregulation of circulating lipids as sensitive biomarkers of IR exposure and DEARE [42,43]. Across both matrices, common changes included perturbations in phospholipids (PCs, PEs, PAs), glycerolipids (DAGs, TAGs), sphingolipids (sphingomyelins and ceramide-related species), fatty acyls (PUFAs, acylcarnitines), amino acids, nucleotide/redox cofactors, and bile acid-related metabolites. In the acute phase, many phospholipids, glycerolipids, and PUFAs declined with concurrent increases in oxylipins and selected acylcarnitines, consistent with widespread membrane injury, lipid peroxidation, and a transient surge in β-oxidation. In the delayed phase, a second pattern emerged, characterized by persistent or recurrent abnormalities in phospholipids, sphingolipids, acylcarnitines, oxylipins, and tryptophan/nicotinamide metabolites, indicating incomplete resolution of redox stress, and evolving DEARE-like mitochondrial and peroxisomal dysfunction. The accumulation of oxylipins and other lipid peroxidation products points to a prominent role for redox-sensitive lipid metabolism in IR-induced injury. These reactive species can affect DNA, proteins, and membranes [44], suggesting that systemic lipid oxidation and disrupted lipid balance may continue well beyond the initial exposure.

EVs displayed distinct features compared with plasma in both acute and delayed phases. While many lipid classes changed in parallel, EV sphingomyelins and acylcarnitines showed unique longitudinal trends: EV sphingomyelins decreased sharply at day 1 but increased by day 90, unlike their plasma counterparts. The early decrease is consistent with localized activation of the sphingomyelinase–ceramide axis in irradiated cells, whereas the later increase, together with enrichment of β-oxidation and peroxisome-related lipids, suggests compensatory reconversion of ceramide to sphingomyelin and sustained export of mitochondrial/peroxisomal stress products. Ceramides are established biomarkers and mediators of IR injury whose accumulation may disrupt lipid rafts and signaling, contributing to diverse post-irradiation pathologies [45,46]. The late increase in EV sphingomyelins at day 90 may indicate enhanced sphingomyelin synthase activity that reconverts ceramide to SM in the setting of inflammatory stress [47]. Such a response may represent a compensatory, anti-apoptotic mechanism disseminated via EVs to buffer ceramide-driven tissue injury [48]. Thus, while plasma reflects the global systemic burden of dyslipidemia and redox imbalance, EVs preferentially capture early ceramide-linked membrane remodeling and later packaging of β-oxidation and peroxisome-derived species. More broadly, the predominance of unique and persistent metabolic perturbations in EV cargo at day 90 underscores EVs as central participants in the propagation and modulation of DEARE. Packaging and export of specific metabolites from irradiated cells into EVs may facilitate their delivery to distant organs, contributing to oxidative stress and degenerative processes at remote sites.

Pathway analysis showed coordinated disruption of interconnected metabolic networks rather than isolated metabolites. In both plasma and EVs, glycerophospholipid and glycosphingolipid metabolism, fatty acid activation and mitochondrial/peroxisomal β-oxidation, the carnitine shuttle, bile acid biosynthesis, and tryptophan/kynurenine metabolism were consistently affected across acute and delayed phases. Together with disturbances in thiamine and nicotinate/nicotinamide pathways, these changes indicate impaired membrane remodeling, mitochondrial and peroxisomal function, and gut–liver–immune crosstalk, along with reduced NAD(H)/NADP(H)-generating capacity. The resulting shift in lipid catabolism favors sustained ROS production and accumulation of lipotoxic intermediates when β-oxidation and the carnitine shuttle are overloaded or inefficient. Concurrent dysregulation of glycerophospholipid and glycosphingolipid metabolism may alter the pool of oxidizable membrane lipids, increasing susceptibility to lipid peroxidation and ferroptosis-like processes, and may generate oxidized bioactive lipids that propagate inflammation and fibrosis. Disruption of bile acid biosynthesis may further interfere with FXR/TGR5 signaling, which is thought to provide antioxidant, cytoprotective, and anti-inflammatory effects in the liver and gut, while altered tryptophan metabolism may reflect breakdown of the gut–liver–immune axis and loss of redox-active, microbiota-derived indole metabolites. Unique pathway changes restricted to either plasma or EVs suggest compartment-specific roles, with EVs preferentially packaging and exporting β-oxidation and peroxisome-related lipids and enzymes as intercellular stress signals, whereas plasma may capture the net systemic metabolic burden. Pathway dysregulation followed a clear temporal pattern: bile acid biosynthesis and glycerophospholipid metabolism were persistent across nearly all time points in both matrices, tryptophan and Vitamin-B metabolism were restricted to the acute phase, and fatty acid β-oxidation, the carnitine shuttle, and glycosphingolipid/ceramide metabolism emerged from day 30 onwards in EVs, coinciding with the compensatory sphingomyelin and β-oxidation lipid changes described above and marking EVs as preferential carriers of delayed, DEARE-associated stress signals. This early-resolving, persistent, and delayed-EV-enriched structure indicates that IR drives temporally distinct waves of metabolic disruption rather than a single fading injury, defining the trajectory from ARS into DEARE.

APC administration partially alleviated these IR-induced disturbances in a time- and APC variant-dependent manner. Across all three APC variants, the pathways most consistently improved in both plasma and EVs were the carnitine-dependent fatty acid transport and β-oxidation, glycerophospholipid and sphingolipid metabolism, nucleotide/redox cofactor metabolism, and amino acid/one-carbon metabolism. The apparent normalization of β-oxidation intermediates, including acylcarnitines and TCA-linked metabolites, may indicate mitigation of mitochondrial and peroxisomal dysfunction, along with reduced ROS and lipotoxicity. The distinct temporal and compartment-specific patterns suggest that individual APC variants differentially engage acute versus delayed phases of IR injury, with implications for targeting ARS and DEARE. Rat 3K3A APC primarily exerted early effects, with normalization by day 14 of key plasma phospholipids and phenylalanine and recovery of EV markers of β-oxidation and redox metabolism, indicating rapid stabilization of membrane and mitochondrial function with modest residual benefits at day 90. This may suggest that 3K3A APC not only stabilizes cellular energy and membrane homeostasis but may also modulate vesicle-mediated export of metabolic stress signals [49]. Rat WT APC produced a more gradual and durable response, with early EV correction of lipid and redox cofactors followed by sustained normalization of plasma kynurenine pathway, sphingolipid/phospholipid, and central carbon metabolites through days 30–90, consistent with prolonged control of oxidative and inflammatory stress. Human WT APC may preferentially modulated amino acid and one-carbon metabolism early and improved mitochondrial, acylcarnitine, sphingomyelin, and ECM-related metabolites later, in both plasma and EVs, suggesting long-term support of energy metabolism. Together, these data indicate that APC variants may act at the pathway level to restore fatty acid oxidation, membrane lipid balance, and redox/one-carbon metabolism, with differing temporal profiles relevant to the early and delayed phases of IR injury. These profiles imply that APC-based countermeasures could be tailored by variant and dosing schedule to more effectively target acute versus delayed IR injury.

The APC-mediated normalization of redox and lipid pathways is consistent with literature reports regarding APC’s antioxidant, anti-inflammatory, and barrier protective actions in sepsis, ischemia–reperfusion, and vascular injury models [38]. Our work extends these concepts into the IR setting, highlighting that APC not only mitigates acute oxidative damage but may also provide a durable, antioxidant-leaning metabolic state reflected in both plasma and EV signatures. Some limitations of this study should be noted. First, the use of a single high-dose exposure limits extrapolation to fractionated, clinically relevant regimens. Second, only female WAG/RijCmcr rats were used, and potential sex-specific differences in IR-induced metabolic responses and APC-mediated IR mitigation were not evaluated, warranting investigation in both sexes in future work. Third, EV isolation is more labor-intensive and currently less scalable than direct plasma analysis, which may limit immediate translation to high-throughput clinical or emergency settings where simpler plasma-based assays are often more practical. Nonetheless, our data underscore that EVs can carry metabolic and lipid signatures that are not readily detectable in bulk plasma, highlighting the unique biological and translational value of EV-focused analyses, particularly in scenarios where detecting very low-abundance or labile cargo, or resolving subtle and compartmentalized lipid perturbations that are diluted or masked in plasma, is critical. Finally, the antioxidant effects of APC are inferred from metabolomic patterns rather than directly measured.

## 5. Conclusions

Overall, this study demonstrates that exposure to IR promotes time-dependent metabolic and lipidomic changes in both plasma and plasma-derived EVs, with EVs carrying distinct and unique signatures. The delayed metabolic alterations in EV, particularly at day 90, indicate that EV cargo is a sensitive marker of delayed radiation responses. Early APC-associated changes in EV pathways (day 14) compared with later changes in plasma (day 90) further suggest that EVs respond rapidly to both radiation and intervention. The relative increase in sphingomyelins in EVs compared to plasma is consistent with the redistribution of sphingomyelin species through EV-mediated communication, potentially counterbalancing ceramide-linked stress signaling. Together, these findings support the use of EV-specific metabolic and lipidomic perturbations as a complementary matrix for radiation biomarker discovery and for understanding systemic adaptations after exposure. The findings from this rat model may be relevant to other mammalian systems, including humans, and provide a basis for further validation of EV-associated metabolic signatures as potential radiation biomarkers. Future studies are needed to define the mechanistic contribution of EV cargo to DEARE and validate EV-derived signatures as predictive biomarkers across additional radiation models.

## Data Availability

All data supporting the findings of this study are provided in the article and the Supplementary Information file.

## Supplementary Materials

The following supporting information can be downloaded at: https://www.mdpi.com/article/10.3390/antiox15091160/s1. This section only contains the caption for Supplementary Tables S1–S9 and Supplementary Figures S1–S5. Supplementary Table S1. List of longitudinally dysregulated metabolites in rat plasma following exposure to 13.0 Gy of X-ray radiation from targeted metabolomics and lipidomics data analysis. Supplementary Table S2. List of longitudinally dysregulated metabolites in rat plasma following exposure to 13.0 Gy of X-ray radiation from untargeted metabolomics and lipidomics profiling. Supplementary Table S3. List of longitudinally dysregulated metabolites in rat plasma derived extra cellular vesicles following exposure to 13.0 Gy of X-ray radiation from targeted metabolomics and lipidomics data analysis. Supplementary Table S4. List of longitudinally dysregulated metabolites in rat plasma derived extracellular vesicles following exposure to 13.0 Gy of X-ray radiation from untargeted metabolomics and lipidomics profiling. Supplementary Table S5. Tandem MS validations for dysregulated metabolites in in rat plasma following exposure to 13.0 Gy of X-ray radiation. Supplementary Table S6. Tandem MS validations for dysregulated metabolites in in rat plasma derived extra cellular vesicles following exposure to 13.0 Gy of X-ray radiation. Supplementary Table S7. List of longitudinally dysregulated pathways in rat plasma following exposure to 13.0 Gy of X-ray radiation from untargeted metabolomics (A) and lipidomics (B) data analysis. Supplementary Table S8. List of longitudinally dysregulated pathways in rat plasma derived Extracellular vesicles following exposure to 13.0 Gy of X-ray radiation from untargeted metabolomics (A) and lipidomics (B) data analysis. Supplementary Table S9. Nanoparticle Tracking Analysis—Camera settings. Figure S1. Panel A. Nanoparticle tracking analysis (NTA) data of isolated EV samples using size-exclusion chromatography showing size-distribution (mean ± SE) and EV concentration (particles/mL). Total particles were calculated by multiplying raw NTA concentration reading by dilution factor (yielding undiluted particles/mL). Panel B. Immunoblot arrays validation expression of known EV markers from rat plasma EV samples isolated using SEC. Figure S2. Longitudinal dyslipidemia profiles in plasma (Panel A) and plasma-derived EVs (Panel B) as revealed by targeted lipidomics analysis, illustrating time-dependent alterations in the lipid species following irradiation and highlighting matrix-specific differences in lipid perturbations that are closely linked to radiation-induced oxidative stress. Figure S3: Quality control assessment of plasma and extracellular vesicle (EV) targeted metabolomics and lipidomics mass spectrometry data. Principal component analysis (PCA) score plots illustrating sample clustering and separation based on targeted metabolite and lipid profiles in EVs and plasma, organized by data acquisition sequence, sample type, and internal standard stability. Distinct grouping of quality control (QC) samples across panels indicates analytical reproducibility and stability over the course of the batch, with no acquisition sequence effect. Panels A–D: EV lipidomics; E–H: EV metabolomics; I–L: plasma lipidomics; M–P: plasma metabolomics. Figure S4: Volcano plots showing dysregulated metabolites in plasma (A–D) and extracellular vesicles (E–F) at 1 day, 14 days, 1 month, and 3 months post-irradiation. Each dot represents a metabolite feature. Grey dots indicate non-significant changes; red dots indicate features that are significantly altered based on both FDR-adjusted *p*-value (< 0.05) and ≥2.0-fold change when comparing irradiated versus sham samples, as determined by targeted mass spectrometry. Figure S5. Box plot representation showing reversal of metabolic dysregulation in rat plasma following 13 Gy X-irradiation in APC-treated animal groups. Panels A–D: rat 3K3A APC at day 90; Panels E–H: rat WT APC at day 14; Panels I–L: human WT APC at day 14.

## Abbreviations

The following abbreviations are used in this manuscript:

**Abbreviation**	**Full term**
ACE	Angiotensin-converting enzyme
ALIX	ALG-2-interacting protein X
APC	Activated protein C
APCHi	Mice with genetically elevated activated protein C (“APC high”)
ARS	Acute radiation syndrome
CD63	Cluster of differentiation 63
CD81	Cluster of differentiation 81
CoA	Coenzyme A
DAG	Diacylglycerol
DCER	Dihydroceramide
DEARE	Delayed effects of acute radiation exposure
DPBS	Dulbecco’s phosphate-buffered saline
ECM	Extracellular matrix
EpCAM	Epithelial cell adhesion molecule
EPCR	Endothelial protein C receptor
EV(s)	Extracellular vesicle(s)
FDA	Food and Drug Administration
FDR	False discovery rate
FFA	Free fatty acid
Flot1	Flotillin-1
FXR	Farnesoid X receptor
Gy	Gray (unit of absorbed radiation dose)
HCER	Hexosylceramide
HMDB	Human Metabolome Database
IR	Ionizing radiation
IACUC	Institutional Animal Care and Use Committee
IPO	Isotopologue Parameter Optimization
IV	Intravenous
LC-MS	Liquid chromatography–mass spectrometry
LC-MS/MS	Liquid chromatography–tandem mass spectrometry
LCER	Lactosylceramide
METLIN	Metabolite mass spectral database (Metabolite Link)
MRM	Multiple reaction monitoring
NAD	Nicotinamide adenine dinucleotide
NADH	Reduced nicotinamide adenine dinucleotide
NADP	Nicotinamide adenine dinucleotide phosphate
NADPH	Reduced nicotinamide adenine dinucleotide phosphate
NHP	Nonhuman primate
NIST	National Institute of Standards and Technology
NTA	Nanoparticle tracking analysis
PAR-1 (PAR1)	Protease-activated receptor-1
PA	Phosphatidic acid
PC	Phosphatidylcholine
PE	Phosphatidylethanolamine
PBI	Partial-body irradiation
PI	Phosphatidylinositol
PUFAs	Polyunsaturated fatty acids
ROS	Reactive oxygen species
SEC	Size-exclusion chromatography
SM	Sphingomyelin
SMs	Sphingomyelins
TCA	Tricarboxylic acid (cycle)
TSG101	Tumor susceptibility gene 101
WT	Wild-type
XCMS	LC–MS data processing software (XCMS)
CEU Mass Mediator	Central European University Mass Mediator (metabolite annotation tool)
