# Supplementary material for: Plasma-Derived Extracellular Vesicles as Systemic Mediators of Radiation Response and Radiation Mitigation by Activated Protein C in a Rat Model
Source: Antioxidants (Basel). 2026 Sep 11;15(9):1160. doi: 10.3390/antiox15091160 (PMC13603334; doi:10.3390/antiox15091160)

Plasma-Derived Extracellular Vesicles as Systemic Mediators of Radiation Response and Radiation Mitigation by Activated Protein C in a Rat Model

Shivani Bansal [1], Sunain Deol [1], Meth Jayatilake [1], Yaoxiang Li [1], Brian L. Fish [2], Xiao Xu [3], Jose A. Fernandez [3], John H. Griffin [3], Tracy Gasperetti [2], Meetha Medhora [2], Marjan Boerma [4], Heather A. Himburg [2] and Amrita K. Cheema [1,5,6,*]

[1] Department of Oncology, Georgetown University Medical Center, Washington, DC 20007, USA; sm3451@georgetown.edu (S.B.); skd74@georgetown.edu (S.D.); mmj61@georgetown.edu (M.J.); yl814@georgetown.edu (Y.L.)
[2] Department of Radiation Oncology, Medical College of Wisconsin, Milwaukee, WI 53226, USA; bfish@mcw.edu (B.L.F.); tgasperetti@mcw.edu (T.G.); medhoram@mcw.edu (M.M.); hhimburg@mcw.edu (H.A.H.)
[3] Department of Translational Medicine, Scripps Research Institute, La Jolla, CA 92037, USA; xxu@scripps.edu (X.X.); jfernand@scripps.ed (J.A.F.); jgriffin@scripps.edu (J.H.G.)
[4] Division of Radiation Health, Department of Pharmaceutical Sciences, University of Arkansas for Medical Sciences, Little Rock, AR 72205, USA; mboerma@uams.edu
[5] Department of Biochemistry and Molecular & Cellular Biology, Georgetown University Medical Center, Washington, DC 20007, USA
[6] Department of Radiation Medicine, Georgetown University Medical Center, Washington, DC 20007, USA
* Correspondence: akc27@georgetown.edu

Extracellular vesicles

Isolation: Plasma samples (150 µL) were aliquoted into 1 mL microcentrifuge tubes and centrifuged at 2,500 x g for 10 minutes at 25 °C. Supernatant was collected and filtered/concentrated through a 100 KDa filter (#UFC510096, Millipore Sigma) by centrifugation at 14,000 x g for 5 minutes at 25 °C. Concentrated plasma sample was collected by inverting the filter into a clean microcentrifuge tube and centrifuging at 1,000 x g for 2 minutes. The recovered volume of concentrated plasma samples varied between 55-95 µL. These volumes were adjusted to a final volume of 150 µL using 0.1 µm filtered 1x PBS and loaded on the SEC columns. Samples were fractionated using 70 nM qEV single columns (SP2, Izon Science) using an automated fraction collector in 200 µL fractions (Izon Science). First three fractions that contain most of the EVs were combined, frozen at -80 °C and lyophilized. Samples were then resuspended in 75 µL 1x PBS and stored at -80 °C until further use.

Characterization

1. **Nanoparticle Tracking Analyses (NTA):** NTA was used to determine the concentration and size distribution of EVs isolated from plasma samples. NTA was performed using a NanoSight NS300 (Malvern Panalytical) equipped with a high sensitivity sCMOS camera, automatic syringe pump and 532 nm laser. 5 µL of EV samples resuspended in 1x PBS were thawed on ice and further diluted to 1 mL with 1x PBS prior to injection. Camera and detection settings are provided in Supplementary Table T9. Videos were captured and processed using NTA 3.3 Dev Build 3.3.104 (Malvern) with 3 videos of 60 seconds per measurement. All samples were analyzed with automatic syringe movement set to 100. The number of EV particles/mL (concentration) and total EV particles/sample (yield) were calculated based on dilution applied and the total working solution obtained.
2. **EV immunoblot array:** To characterize the isolated EVs and confirm the isolation of bonafide EVs, we performed the Exo-check Antibody Array (System Biosciences, Palo Alto CA, #EXORAY210A) and examined the expression of classical EV-specific protein markers (TSG101, CD63, CD81, EpCAM, ALIX, Annexin5, Flotilin1, ICAM). EVs resuspended in 1x PBS were thawed on ice, and total protein was quantified by a bicinchoninic acid assay. EV samples consisting about 30 µg of protein were aliquoted. Immunoblots were processed and developed according to the manufacturer's protocol. A detailed characterization of the EV preparations was performed in accordance with MISEV guidelines. The examined EV-specific protein markers included the transmembrane proteins such as CD63 and CD81 as well as cytosolic proteins like TSG101, ALIX in the plasma EVs.

Mass Spectrometry Protocols

Untargeted Plasma and Plasma EV Metabolomics and Lipidomics Using UPLC-QToF-MS

Sample preparation for plasma: Plasma samples were taken out of storage at −80 °C and thawed on ice. For extraction, 75 µL of chilled extraction buffer that was prepared by mixing water:methanol:isopropanol (35:25:40) and containing internal standards (de-brisoquine and 4-nitrobenzoic acid) was added to 25 µL of plasma sample. The samples were vortexed and incubated on ice for 20 min. Next 100 µL of chilled acetonitrile was added, vortexed and incubated at −20 °C for 20 minutes. Finally, the samples were centri-fuged at 13,000 rpm for 20 minutes at 4 °C. The supernatants were transferred to MS vials for LC-MS data acquisition. Then, 20 µL from each sample was mixed to generate a pooled QC sample.

Sample preparation for plasma EV: The EV samples (45 µL resuspended in 1× PBS) stored at −80 °C were thawed, placed on dry ice for 30 s and heat shocked by plunging them into a 37 °C water bath

for 90 s. This cycle was repeated two more times followed by sonication for 1 min and then incubation on ice for 20 min. Next, 135 μL of chilled extrac-tion buffer, prepared by mixing water:methanol:isopropanol (35:25:40) containing internal standards (debrisoquine and 4-nitrobenzoic acid), was added. Samples were then vor-texed and kept on ice for 20 min. Next, 135 μL of chilled acetonitrile was added and sam-ples were vortexed again for 30 s followed by incubation at −20 °C for 20 min. Finally, samples were centrifuged at 13,000× g for 20 min at 4 °C and the supernatants were trans-ferred to MS vials for LC-MS data acquisition. Then, 20 μL from each sample was mixed to generate a pooled QC sample.

Data acquisition: The sample queue was randomized to avoid bias. For metabolomics data acquisition, each sample (1 μL) was injected to a 130Å, 1.7 μm, 2.1 mm × 50 mm Ac-quity BEH C18 column (Waters Corporation, Milford, MA, USA) using an Acquity UPLC system connected to an electrospray ion source coupled with a quadrupole time-of-flight mass spectrometer (ESI-Q-TOF, Xevo-G2S, Waters Corporation, MA, USA) operating in positive and negative ionization mode. The gradient mobile phases consisted of 100% wa-ter with 0.1% formic acid (solvent A), 100% acetonitrile with 0.1% formic acid (solvent B), 100% isopropanol with 0.1% formic acid (solvent C). Each sample injection was run for 13 min at a flow rate for 400 μL/min. The LC gradient conditions with a ramp curve of 6 at each step were as follows: Initial—98% A, 2% B till 0.5 min; 4.0 min—40% A, 60% B; 8.0 min—2% A, 98% B till 9.0 min; 9.5 min—11.8% B, 88.2% C till 11 min; 11.5 min—50% A, 50% B, 12.0 min—98% A, 2% B till 13 min. For lipidomics data acquisition, each sample (1 μL) was injected to a 1.7 μm, 2.1 mm × 100 mm Acquity CSH C18 column (Waters Cor-poration, MA, USA) using an Acquity UPLC system connected to an electrospray ion source coupled with a quadrupole time-of-flight mass spectrometer (ESI-Q-TOF, Xevo-G2S, Waters Corporation, MA, USA) operating in positive and negative ionization mode. The gradient mobile phase consisted of 100% water with 0.1% formic acid and 10 mm ammo-nium formate (solvent A), 100% acetonitrile with 0.1% formic acid (solvent B), 100% iso-propanol with 0.1% formic acid (solvent C). Each sample injection was run for 11 min at a flow rate for 450 μL/min. The LC gradient conditions with a ramp curve of 6 at each step were as follows: Initial—30% A, 34% B, 36% C till 0.5 min; 8 min—0% A, 10% B, 90% C till 8.5 min; 9.0 min—30% A, 34% B, 36% C till 11 min.

MS parameters: In the MS method, positive mode had a capillary voltage of 3.0 kV, a sampling cone voltage of 30 V, and a source offset of 80 V. Negative mode had a capillary voltage of 2.0 kV, a sampling cone voltage of 40 V (30 V for lipidomics), and a source offset of 80 V. The desolvation gas flow was 1000 L/h and the temperature was set to 500 °C. The cone gas flow was 25 L/h and the source temperature was 120 °C. The data were acquired in the sensitivity MS Mode with a scan time of 1.000 seconds, and inter-scan delay at 0.014 seconds.

The data were acquired in centroid TOF-MS mode over a mass range from 50 to 1200 m/z. Accurate mass was maintained by infusing Leucine Enkephalin (556.2771 m/z) in 50% aqueous acetonitrile (0.5 ng/mL) at a rate of 20 μL/min via the Lockspray interface for real-time mass correction, every 10 s. Before and after samples run, a mixture of six standards (acetaminophen: m/z 152.0712 $[M+H]^+$/150.0555 $[M-H]^-$, sulfaguanidine: m/z 215.0603 $[M+H]^+$/213.0446 $[M-H]^-$, sulfadimethoxine: m/z 311.0814 $[M+H]^+$/309.0658 $[M-H]^-$, Val-Tyr-Val: m/z 380.2185 $[M+H]^+$/378.2029 $[M-H]^-$, terfenadine: m/z 472.3216 $[M+H]^+$ and leucine-enkephalin: m/z 556.2771 $[M+H]^+$/554.2615 $[M-H]^-$) were run to en-sure mass accuracy during data acquisition. Several measures were used to ensure the high quality and reproducibility of LC-MS data. The column was conditioned using pooled QC samples, which were injected periodically through the batch to monitor mass accuracy, shifts in retention time and signal intensities as measures of reproducibility. The overlap of QC sample chromatograms (base peak intensity) shows minimal shifts in reten-tion

time and consistency in peak intensities throughout the acquisition. Pooled QC sam-ples injected routinely were also used to assess and correct for any analytical variance.

UPLC-QToF-MS data pre-processing: The MS generated raw data files were first con-verted into NetCDF files for pre-processing using Waters MassLynx Databridge Software within MassLynx v4.1 (Waters Corporation, Milform, MA, USA). XCMS was used for pre-processing the data files while the Isotopologue Parameter Optimization (IPO) pack-age Version 1.14.0 (open-source bioinformatics software package developed by researchers at JOANNEUM RESEARCH Forschungsgesellschaft mbH, Graz, Styria, Austria) was used for XCMS parameter optimization [29]. Normalization was performed with internal standards in both positive and negative mode data. The features with more than 20% missing value were also filtered out and the features with less than 20% of missing value were imputed by half of the minimum positive value in the original data. The features with more than 25% of coefficient of variation (CV) were also filtered out. The remaining high-quality features were normalized by QC-RLSC [30]. R package was used to perform multivariate analysis [31]. Statistically significant m/zs with FDR adjusted p-value < 0.05 were run on the UPLC-QToF instrument (Waters Corporation, Milford, MA, USA) in the MS/MS mode. The MS/MS raw data files were converted to NetCDF files using Waters MassLynx Databridge Software, then further converted to MSP file format using an in-house R package. Thereafter, for accurate mass-based putative identification of metabo-lites, we performed database search by matching MS/MS spectra to entries in the NIST 2017 MS/MS database and by applying the online version of CEU Mass Mediator (CMM) (an open-access bioinformatics web tool, developed and maintained by the Centre for Metabolomics and Bioanalysis (CEMBIO) at Universidad San Pablo-CEU, Madrid, Ma-drid, Spain) which integrated from METLIN, Human Metabolome Database (HMDB) and LIPID MAPS with a ppm error of less than 10.

Targeted Metabolomics and Lipidomics Using Sciex 5500 QTRAP

We employed in-house-developed targeted multiple reaction monitoring (MRM)-based quantitative lipidomics and metabolomics analytical methodologies. For this purpose, all LC-MS-grade solvents, including acetonitrile and water, were purchased from Fisher Optima-grade, Fisher Scientific. High-purity formic acid (99%) was purchased from Thermo-Scientific (Waltham, MA, USA). Debrisoquine and 4-nitrobenzoic acid were purchased from Sigma-Aldrich. EquiSPLASH® LIPIDOMIX® quantitative mass spec inter-nal standard and 15:0–18:1-d7-PA, C15 Ceramide-d7 (d18:1-d7/15:0), and 18:1 Chol (D7) ester were purchased from Avanti Polar Lipids (Alabaster, AL, USA). Internal standards for free fatty acids (FFAs), dihydroceramides (DCER), hexosylceramides (HCERs), and lactosylceramides (LCERs) were purchased from Sciex as Lipidyzer platform kits. All samples were randomized, processed and analyzed using standardized procedures to minimize potential technical variation. Details of the sample preparation, LC-MS condi-tions, data acquisition, data pre-processing, and statistical analyses have been detailed below.

Plasma sample preparation: Polar plasma metabolites and lipids were extracted us-ing a protein precipitation-based protocol optimized for LC–MS/MS analysis. For this purpose, 25 μL of each plasma sample was mixed with 125 μL of chilled isopropanol containing internal standards. The samples were vortexed for 1 min and kept on ice for 30 min, then incubated at −20 °C for 2 h to allow protein precipitation. Subsequently, the samples were centrifuged at 13,000 rpm for 20 min at 4 °C. The resulting supernatant was transferred to MS vials for LC–MS analysis. To generate a pooled quality control (QC) sample, 20 μL of each prepared sample was combined.

Plasma EV sample preparation: Polar EV metabolites and lipids were extracted using a protein precipitation-based protocol optimized for LC–MS/MS analysis. For this purpose, 15 μL of each EV sample was mixed with 40 μL of a water/methanol/isopropanol mixture (35:25:40, v/v/v). The sample tubes were subjected to three freeze–thaw cycles by alternat-ing immersion in dry ice for 30 s and a 37 °C water bath for 90 s, followed by sonication for 1 min. Subsequently, 100 μL of chilled

isopropanol containing internal standards was added to each sample. The samples were vortexed for 1 min and kept on ice for 30 min, followed by incubation at −20 °C for 2 h to facilitate protein precipitation. The samples were then centrifuged at 13,000 rpm for 20 min at 4 °C, and the resulting supernatant was transferred to MS vials for LC–MS analysis. To generate a pooled QC sample, 20 μL of each prepared sample was combined.

Targeted metabolomics acquisition: Targeted metabolomics analysis was performed using a QTRAP 5500 LC–MS/MS system (SCIEX, MA, USA) operated in scheduled multi-ple reaction monitoring (MRM) mode with polarity switching to enable detection in both positive and negative ion modes. The targeted assay was developed in-house and enabled quantification of 270 endogenous metabolites spanning multiple biochemical pathways. Chromatographic separation was achieved on a Kinetex F5 column (2.6 μm, 100 Å, 100 × 2.1 mm; Phenomenex, CA, USA) maintained at 30 °C, with a flow rate of 0.200 mL/min. A 5 μL aliquot of each processed sample was injected using a SIL-30AC autosampler (Shi-madzu, Kyoto, Japan), which was coupled to an LC-30AD solvent delivery unit and CBM-20A communication module (Shimadzu, Kyoto, Japan), with the autosampler tem-perature maintained at 15 °C and a 2 s needle wash using a 1:1:1:1 wa-ter/isopropanol/methanol/acetonitrile rinse solvent. The mobile phases consisted of water with 0.1% formic acid (A) and acetonitrile with 0.1% formic acid (B). The gradient was programmed as follows: 0–2.1 min, 100% A; 2.1–14.0 min, linear decrease to 5% A; 14.0–16.0 min, 5% A and 95% B; 16.1–20.0 min, return to 100% A, with a total run (stop) time of 21.0 min and a maximum pressure limit of 15,000 psi. The mass spectrometer was operat-ed with a turbo spray ion source at 400 °C, using curtain gas at 35 psi, heater gas at 60 psi, ion source gas 1 at 60 psi, ion source gas 2 at 70 psi, and nebulizing gas at 50 psi. The ca-pillary voltage was set to +5500 V in positive mode and −4500 V in negative mode, with medium collision-activated dissociation (CAD) gas and an entrance potential of 10 V.

Targeted lipidomics acquisition: Targeted lipidomics analysis was performed using the same QTRAP 5500 LC–MS/MS system configured with class-specific MRM transitions to enable comprehensive coverage and quantification of major lipid subclasses, including phospholipids (phosphatidylcholines, lysophosphatidylcholines, phosphatidic acids, lysophosphatidic acids, phosphatidylethanolamines, lysophosphatidylethanolamines, phosphatidylglycerols, phosphatidylserines, phosphatidylinositols, and lysophosphati-dylinositols), glycerolipids (monoacylglycerols, diacylglycerols, and triacylglycerols), sphingolipids (sphingomyelins, ceramides, hexosylceramides, dihydroceramides, and lactosylceramides), free fatty acids, cholesterol esters, and acylcarnitines. Chromatograph-ic separation was achieved on an XBridge BEH Amide column (100 × 4.6 mm, 3.5 μm; Wa-ters, MA, USA) maintained at 30 °C. The mobile phases consisted of 10 mM ammonium acetate in 95:5 acetonitrile/water (A) and 10 mM ammonium acetate in 50:50 acetoni-trile/water (B). The LC gradient was run at a flow rate of 0.2–0.7 mL/min as follows: 0–3.0 min, 100% A; 3.0–3.05 min, 99.9% A/0.1% B; 3.05–3.10 min, 99.8% A/0.2% B (flow in-creased to 0.7 mL/min); 3.10–6.0 min, ramp to 94% A/6% B; 6.0–10.0 min, ramp to 75% A/25% B; 10.0–11.0 min, 2% A/98% B; 11.0–13.0 min, 0% A/100% B; 13.0–18.6 min, 0% A/100% B at 0.7 mL/min; 18.6–18.8 min, return to 100% A while reducing flow to 0.2 mL/min; 18.8–22.0 min, 100% A at 0.2 mL/min, with a stop time of 23.0 min and a maxi-mum pressure of 18,000 psi. A 2 μL aliquot of each sample was injected using an au-tosampler maintained at 15 °C, with a 2 s needle wash using a 1:1:1:1 wa-ter/isopropanol/methanol/acetonitrile rinse solvent. The mass spectrometer was operated with a turbo spray ion source at 550 °C, using curtain gas at 30 psi, heater gas at 60 psi, ion source gas 1 at 50 psi, ion source gas 2 at 60 psi, and medium collision gas. The capil-lary voltage was set to +5500 V in positive mode and −4500 V in negative mode.

Quality Control and Instrument Performance Monitoring: To monitor analytical re-producibility and instrument stability, a pooled quality control (QC) sample was prepared by combining 10 μL from each processed plasma/EV sample. All samples were maintained at low temperatures during

preparation to minimize metabolite degradation and prevent artifactual changes. Stringent quality control procedures were implemented to ensure high analytical reproducibility and data robustness. Prior to batch acquisition, the chromato-graphic column was conditioned using 5 repeated injections of pooled QC samples to sta-bilize retention times and signal intensities. During the analytical run, pooled QC samples were injected after every 10 study samples to monitor instrumental drift, retention time stability, and signal variation. Blank solvent injections were included after every 10 sam-ples, both before and after QC injections, to evaluate potential sample carry-over and background contamination. Additionally, a NIST standard reference plasma sample was analyzed after every 20 injections to monitor instrument performance and inter-batch con-sistency. Analytical signal drift across the batch was corrected using quality control-based robust locally estimated scatterplot smoothing (QC-RLSC).

Data processing and statistical analysis: The abundance measurement for metabolites and lipids were derived as intensity units that were initially normalized to internal standards and processed using MultiQuant 3.0.3 (SCIEX). The data were pre-processed using a signal/noise ratio >20:1 and retention time (RT) tolerance of 5 s, after manually checking of metabolites peak by experts to find the reliable features. We also use 20% of missing values in each feature as filter-out criteria. Missing values were imputed by half of the minimum positive value in the original data. Thereafter, we used 20% of the coefficient of variation (CV) as our filter criteria to remove any possible noises before data normaliza-tion. Analytical drifts (if any) were corrected by quality control-based robust LOESS signal correction (QC-RLSC). All the analyses were performed in MetaboAnalyst (V5.0) and R (v 4.0.3). The normalized LC-MS data was log-transformed and Pareto-scaled. For the 185 samples in the study set, the level of differential expression for each metabolite was calcu-lated using an unpaired t-test, comparing sham vs. radiation, and radiation vs. radiation plus APC-treated samples, constrained by the FDR-corrected p-value < 0.05. MetaboAna-lyst (V5.0) was used to evaluate the metabolic pathways affected by radiation exposure longitudinally and radioprotective alleviation by APC treatments.

Figure S1. Panel A. Nanoparticle tracking analysis (NTA) data of isolated EV samples using size-exclusion chromatography showing size-distribution (mean ± SE) and EV concentration (particles/mL). Total particles arrived at by multiplying raw NTA concentration reading by dilution factor (yielding undiluted particles/mL). **Panel B.** Immunoblot arrays validation expression of known EV markers from rat plasma EV samples isolated using SEC.

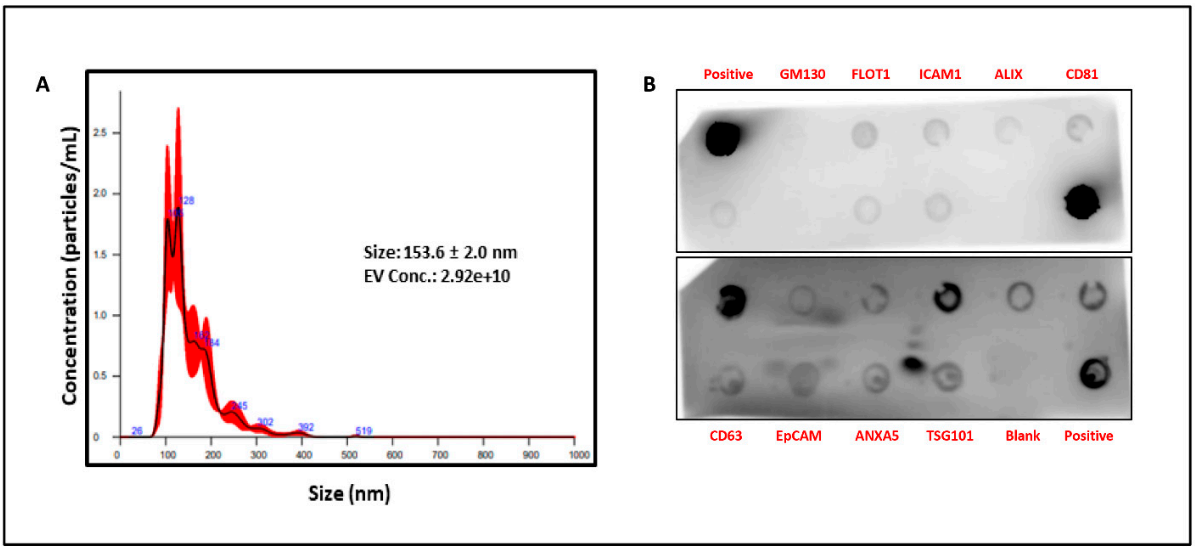

Figure S2. Longitudinal dyslipidemia profiles in plasma (Panel A) and plasma-derived EVs (Panel B) as revealed by targeted lipidomics analysis, illustrating time-dependent alterations in the lipid species following irradiation and highlighting matrix-specific differences in lipid perturbations that are closely linked to radiation-induced oxidative stress.

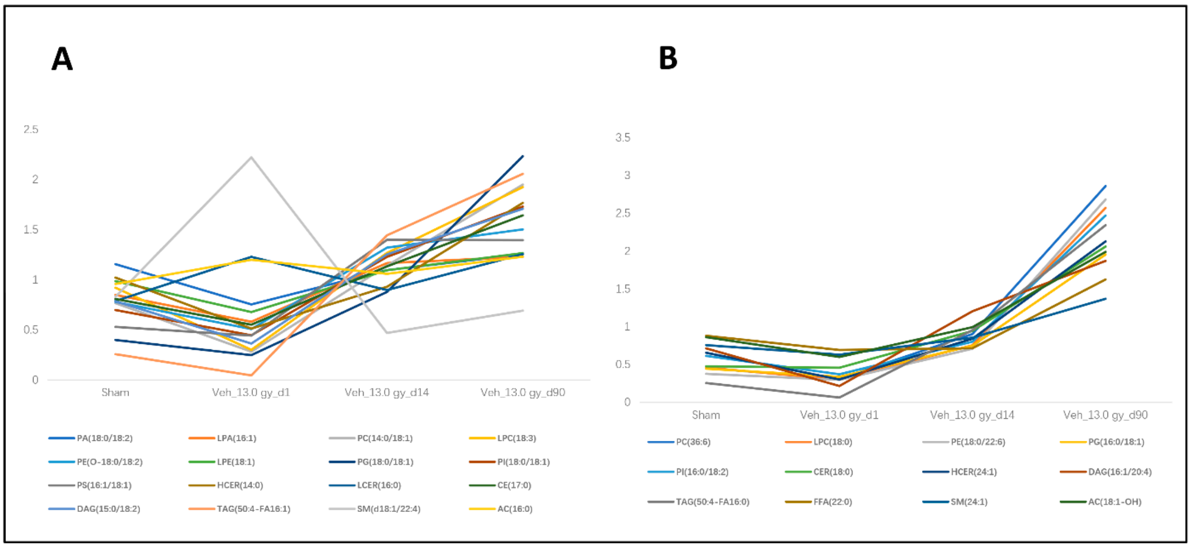

Figure S3: Quality control assessment of plasma and extracellular vesicle (EV) targeted metabolomics and lipidomics mass spectrometry data. Principal component analysis (PCA) score plots illustrating sample clustering and separation based on targeted metabolite and lipid profiles in EVs and plasma, organized by data acquisition sequence, sample type, and internal standard stability. Distinct grouping of quality control (QC) samples across panels indicates analytical reproducibility and stability over the course of the batch, with no acquisition sequence effect. Panels A-D: EV lipidomics; E-H: EV metabolomics; I-L: plasma lipidomics; M-P: plasma metabolomics.

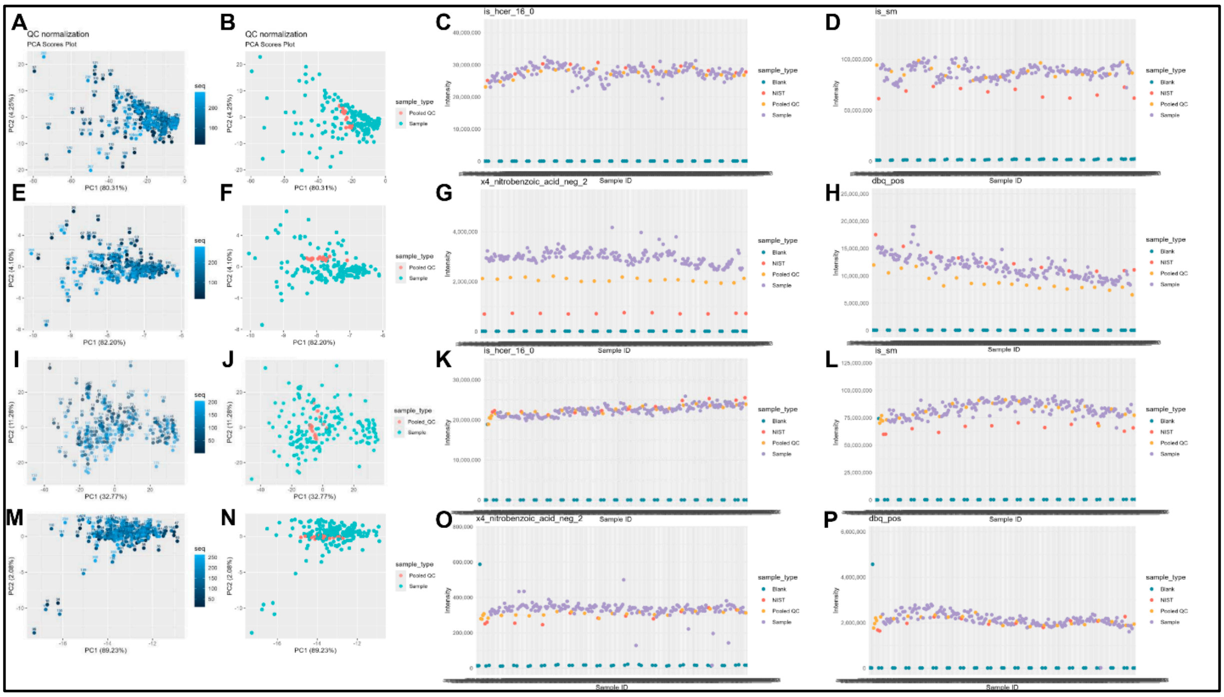

Figure S4: Volcano plots showing dysregulated metabolites in plasma (A–D) and extracellular vesicles (E–F) at 1 day, 14 days, 1 month, and 3 months post-irradiation. Each dot represents a metabolite feature. Grey dots indicate non-significant changes; red dots indicate features that are significantly altered based on both FDR-adjusted p-value (< 0.05) and ≥2.0-fold change when comparing irradiated versus sham samples, as determined by targeted mass spectrometry.

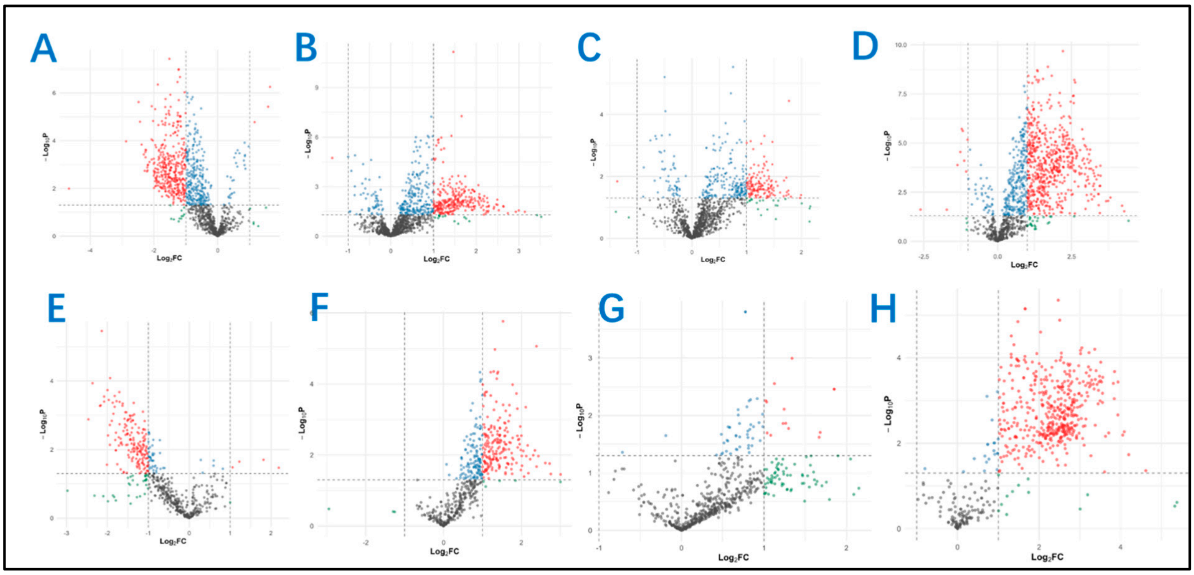

Figure S5. Box plot representation showing reversal of metabolic dysregulation in rat plasma following 13 Gy X-irradiation in APC-treated animal groups. Panels A-D: rat 3K3A APC at day 90; Panels E-H: rat WT APC at day 14; Panels I-L: human WT APC at day 14.

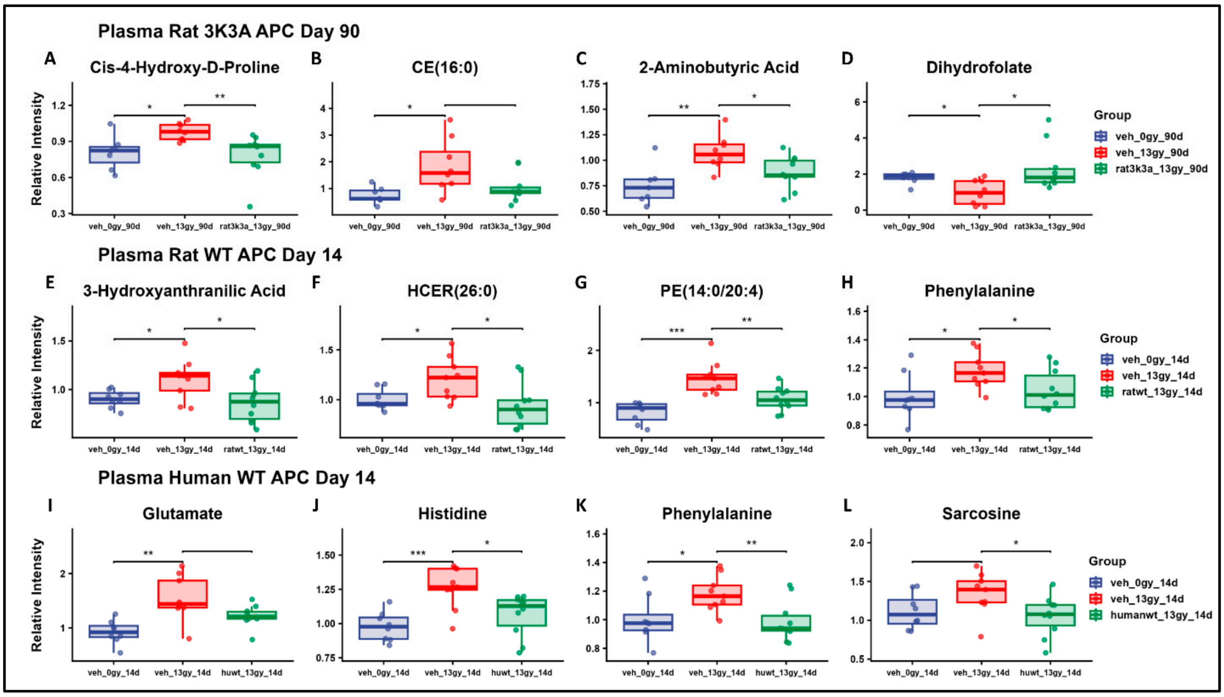

Supplement: Supplementary file 1 [file antioxidants-15-01160-s001.zip › Supplementary File.pdf]
